# Human Tumor-Infiltrating MAIT Cells Display Hallmarks of Bacterial Antigen Recognition in Colorectal Cancer

**DOI:** 10.1016/j.xcrm.2020.100039

**Published:** 2020-06-23

**Authors:** Shamin Li, Yannick Simoni, Etienne Becht, Chiew Yee Loh, Naisi Li, Daniel Lachance, Si-Lin Koo, Teck Por Lim, Emile Kwong Wei Tan, Ronnie Mathew, Andrew Nguyen, Justin Golovato, Julia D. Berkson, Martin Prlic, Bernett Lee, Samuel S. Minot, Niranjan Nagarajan, Neelendu Dey, Daniel S.W. Tan, Iain B. Tan, Evan W. Newell

**Affiliations:** 1 Vaccine and Infectious Diseases Division, Fred Hutchinson Cancer Research Center, Seattle, WA, USA; 2 Agency for Science, Technology and Research (A∗STAR), Singapore Immunology Network (SIgN), Singapore, Singapore; 3 Clinical Research Division, Fred Hutchinson Cancer Research Center, Seattle, WA, USA; 4 Division of Medical Oncology, National Cancer Centre Singapore (NCCS), Singapore, Singapore; 5 Computational and Systems Biology, Genome Institute of Singapore, Singapore, Singapore; 6 Department of Colorectal Surgery, Singapore General Hospital, Singapore, Singapore; 7 NantOmics, Santa Cruz, CA, USA; 8 Microbiome Research Initiative, Fred Hutchinson Cancer Research Center, Seattle, WA, USA; 9 Yong Loo Lin School of Medicine, National University of Singapore, Singapore, Singapore

**Keywords:** MAIT, colorectal cancer, CD39, microbiome, CyTOF

## Abstract

Growing evidence indicates a role for the gut microbiota in modulating anti-tumor treatment efficacy in human cancer. Here we study mucosa-associated invariant T (MAIT) cells to look for evidence of bacterial antigen recognition in human colon, lung, and kidney carcinomas. Using mass cytometry and single-cell mRNA sequencing, we identify a tumor-infiltrating MAIT cell subset expressing CD4 and Foxp3 and observe high expression of CD39 on MAIT cells from colorectal cancer (CRC) only, which we show *in vitro* to be expressed specifically after TCR stimulation. We further reveal that these cells are phenotypically and functionally exhausted. Sequencing data show high bacterial infiltration in CRC tumors and highlight an enriched species, *Fusobacteria nucleatum,* with capability to activate MAIT cells in a TCR-dependent way. Our results provide evidence of a MAIT cell response to microbial antigens in CRC and could pave the way for manipulating MAIT cells or the microbiome for cancer therapy.

## Highlights

Comprehensive profiling of tumor-infiltrating MAIT cells in colorectal cancer (CRC)
Identification of a tumor-infiltrating MAIT cell subset expressing CD4 and Foxp3
TCR-driven CD39 expression is enriched in tumor-infiltrating MAIT cells
CRC-related *Fusobacterium nucleatum* can activate MAIT cells in a TCR-dependent way

## Graphical Abstract

**Figure:**
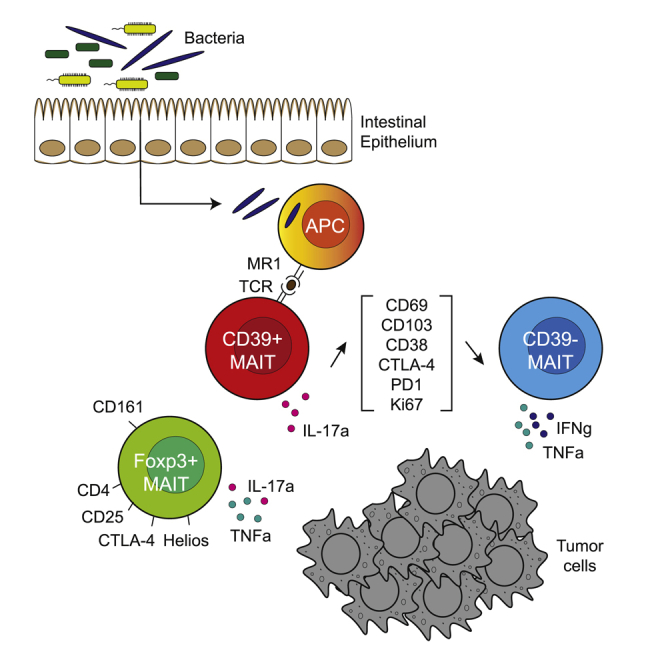

## Teaser

By investigating human colon, lung, and kidney carcinomas, Li et al. propose MAIT cells as part of the missing link between the gut microbiota and tumor biology. This work will open new avenues in cancer therapy by manipulating MAIT cells or MAIT-related bacterial ligands in the context of microbiota-influenced cancers.

## Introduction

The era of cancer immunotherapy is in full swing, and different forms of treatment, such as checkpoint blockade immunotherapy, show uneven effects on restoring T cell immune responses. There is mounting evidence that the gut microbiota can strongly influence the antitumor efficacy of drugs (reviewed in Helmink et al.^1^). The first studies suggesting an immunotherapeutic effect of the microbiome showed improved effects of tumor-specific T cells by transferring bacterial products from the intestinal lumen to secondary lymphoid organs.^2^ Ever since, the composition of the gut microbiota has been reported to influence anti-cancer therapeutic responses, including immune checkpoint blockade therapies targeting CTLA-4 and PD-1 (reviewed in Helmink et al.^1^). However, little is known about the mechanisms behind the varying responses.

Mucosa-associated invariant T (MAIT) cells are part of the unconventional or innate-like T cell family. These cells recognize bacterial antigens presented through MR1, a non-classical major histocompatibility complex (MHC) class I-like molecule. Mainly resident in the mucosa, MAIT cells are also found in lymphoid tissues and organs such as the liver and can account for up to 10% of total T cells in peripheral blood.^3^, ^4^, ^5^, ^6^, ^7^ In humans, MAIT cells express the invariant T cell receptor (TCR) Vα7.2 associated with Jα33, Jα12, or Jα20. The TCR-β chains are more diverse, with a bias toward Vβ2 and Vβ13.^3^^,^^4^^,^^8^^,^^9^ In 2012, identification of riboflavin precursor derivatives as the microbial ligands for MAIT cells allowed synthesis of MR1 tetramers to specifically identify these cells.^10^^,^^11^ Most human MAIT cells are CD8^+^, express the effector/memory phenotype CD45RO^+^ CD62L^lo^ CCR7^−^, and are IL-18R^hi 3^. They are known to protect against bacterial infection, are reported to be depleted during viral infection, and are implicated in several autoimmune diseases, including diabetes.^12^, ^13^, ^14^

In human cancers, the first report documenting the presence of MAIT cells showed that TRAV1-2 (coding for Vα7.2) and TRAJ33 (coding for Jα33) transcripts are enriched in brain and kidney tumors.^15^ Other studies have shown an accumulation of these cells in colon adenocarcinoma with a reduced capability to produce interferon γ (IFNγ) and a positive correlation between a high tumor-infiltrating MAIT cell ratio and poor patient outcome (reviewed in Haeryfar et al.^16^). These results seem to indicate a negative effect of MAIT cell infiltration in tumors, but the function of these cells in the tumor microenvironment is lacking.

Because the riboflavin synthesis pathway is broadly conserved among many species of bacteria, MAIT cells can respond to a wide array of microbes, including known commensals.^10^^,^^17^ Some groups have reported functional heterogeneity of MAIT cells as they respond to different bacterial or fungal organisms and adapt their antimicrobial response patterns.^18^ In this regard, a recent study has assessed the ability of a large variety of commensal bacteria to activate MAIT cells in *in vitro* functional assays through human T cells engineered for MAIT TCRs.^18^ These studies showed a potential effect of bacteria in shaping the function of MAIT cells under pathophysiological conditions. Here we hypothesize that MAIT cell responses can be initiated and modulated by gut microbiome-generated antigens in the tumor microenvironment. We aim to discern the role of MAIT cells at the interface between mucosa-associated cancers and the human gut microbiome by profiling colorectal cancer (CRC), non-small cell lung carcinoma (NSCLC), and renal cell carcinoma (RCC).

## Results

### Tumor-Infiltrating MAIT Cells from CRC Show a Distinct Protein and Gene Profile

**Figure 1:**
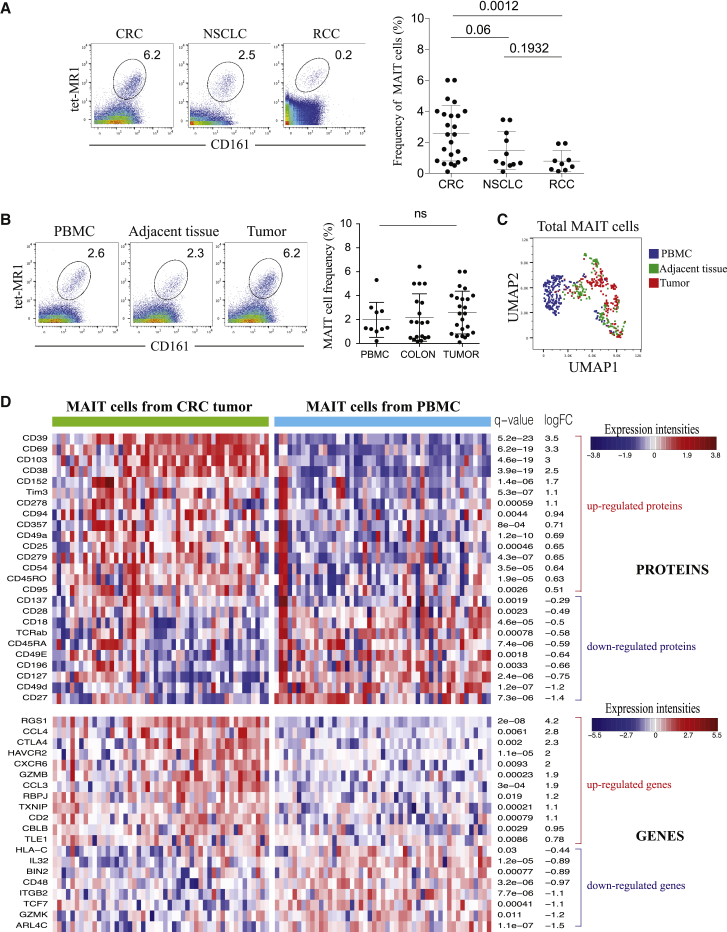
Tumor-Infiltrating MAIT Cells from CRC Show a Distinct Protein and Gene Profile (A) Representative mass cytometry staining of MAIT cells in CRC, NSCLC, and RCC, gated on CD45^+^ live, DNA^+^, CD14^–^CD16^–^ CD3^+^ T cells (left) and frequencies of MAIT cells in the different tumors. CRC = 24, NSCLC = 11, RCC = 9 (right). Data are mean with SD from at least 10 experiments. Mann-Whitney U test. (B) Representative MAIT cell staining from PBMC, adjacent tissue, and tumor of CRC, gated on total T cells. Shown are frequencies of MAIT cells in different compartments. PBMC = 10, colon = 19, tumor = 19. Data are mean with SD from at least 7 experiments. Mann-Whitney U test. (C) UMAP plot of total MAIT cells from 2 PBMCs, 7 adjacent tissues, and 7 tumors of the same experiment. (D) Heatmap showing differences in protein and gene expression between MAIT cells from CRC tumors and control healthy PBMC. Shown are only proteins and genes that are differentially expressed, with a fold-change of ±0.25 or more and adjusted p value (q value) of 0.05 or less. ScRNA-seq combined with the BD AbSeq Rhapsody system on sorted MAIT cells gated on live T cells using MR1 tetramer (STAR Methods). See also Figure S1 and Data S1.

We first analyzed the frequency of MAIT cells in tumor samples from CRC, NSCLC, and RCC patients by mass cytometry (also known as CyTOF; STAR Methods). To ensure the robustness of our 5-OP-RU MR1 tetramer staining, we used Vα7.2 to confirm the specificity of 5-OP-RU MR1 and 6-FP MR1 to verify the absence of unspecific staining (Figures S1A and S1B). We observed that MAIT cells accounted for a higher proportion of total T cells in CRC compared with NSCLC and RCC (Figure 1A). No clear difference was detected in peripheral MAIT cell frequency between the three cancer types, indicating that the high infiltration of MAIT cells in CRC was tumor specific (Figure S1C). Using a 39-parameter panel, we focused our analysis on profiling tumor-infiltrating MAIT cells from CRC compared with PBMC and healthy adjacent tissue used as references. Although no difference was observed in MAIT cell frequency (Figure 1B), our analysis revealed a distinct phenotype of MAIT cells derived from tumor versus adjacent tissue or PBMC^19^ (Figures 1C and S1D). At the gene level, bulk RNA sequencing of sorted MAIT cells showed a distinct transcriptomic profile between blood-circulating and tumor-infiltrating MAIT cells (Figure S1E). Specifically, gene set enrichment analysis (GSEA) highlighted an enrichment of TCR signaling and negative apoptotic regulation pathways from tumor-infiltrating MAIT cells (Figures S1F and S1G; Data S1). To further profile MAIT cells from CRC, we sorted MAIT cells from tumors and performed single-cell targeted mRNA sequencing (scRNAseq) in parallel with protein expression profiling using AbSeq on the BD Rhapsody system (STAR Methods).^20^) MAIT cells from healthy donor (HD) PBMC were analyzed simultaneously as a reference. We confirmed distinct protein and gene profiles in MAIT cells derived from tumors and PBMC (Figure 1D). Tumor-infiltrating MAIT cells highly expressed CD69, CD103, CD38, and CD39 with lower expression of CD27 and CD49d compared with peripheral MAIT cells. At the gene level, most tumor-infiltrating MAIT cells expressed CCL4, CCL3, and RGS1, indicating a high response to inflammation (Figure 1D). Moreover, these data revealed a heterogeneity among tumor-infiltrating MAIT cells from CRC that was not observed in peripheral MAIT cells. For instance, we detected the presence of CD39^+^ and CD39^−^ populations, each expressing a specific protein and transcriptomic signature. In the CD39^+^ population, we also distinguished subsets with unique protein and gene expression (CD69^+^, CD103^+^, and CD38^+^ versus CD152^+^, Tim3^+^, CD357^+^, and CD45RA^+^).

### Identification of a CD4^+^ Foxp3^+^ Subset in Tumor-Infiltrating MAIT Cells

**Figure 2:**
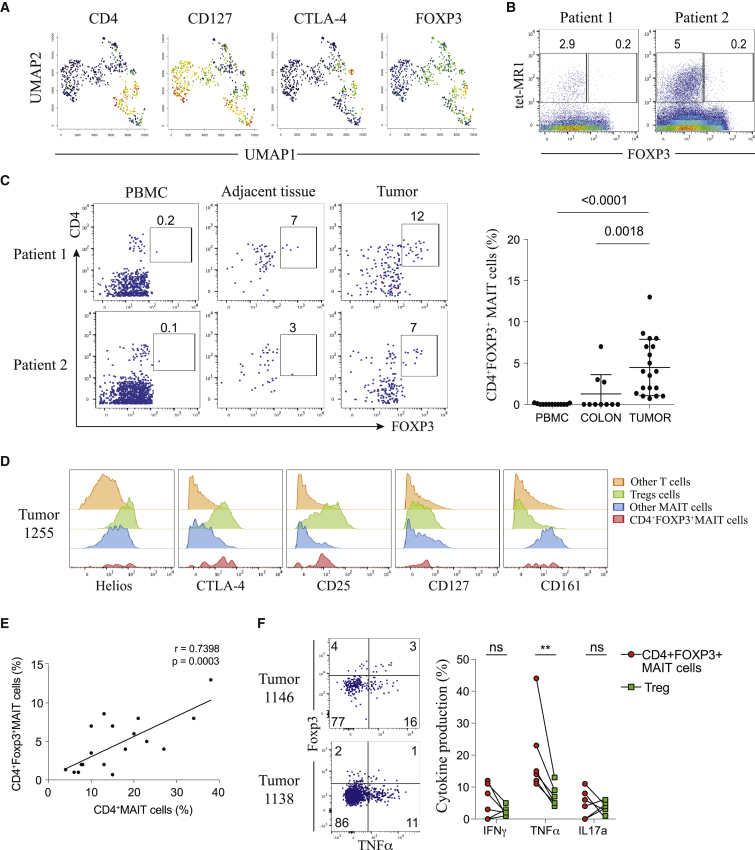
Identification of a CD4^+^ Foxp3^+^ Subset in Tumor-Infiltrating MAIT Cells (A) UMAP plots showing expression of selected markers on MAIT cells; intensities are red (high), yellow/green (intermediate), blue (low). (B) Tet-MR1 staining plotted against Foxp3 on total T cells from two CRC tumor samples. (C) Representative staining of CD4^+^ Foxp3^+^ MAIT cells from PBMC, adjacent tissue, and tumors of two patients, gated on total MAIT cells. Shown are frequencies of Foxp3 expression among total MAIT cells (PBMC = 13, colon = 10, tumor = 19). Data are mean with SD from at least 7 experiments. Mann-Whitney U test. (D) Expression intensities of Treg-related markers and CD161 on different T cells compared with the CD4^+^ Foxp3^+^ MAIT subset; one representative tumor sample. (E) Correlation of Foxp3 expression on MAIT cells with CD4^+^ MAIT cell frequency; n = 20, two-tailed paired t test, Pearson’s correlation. (F) Co-expression of Foxp3 and TNF-α gated on total MAIT cells (left) and cytokine production (IFNγ, TNF-α, and IL-17) by CD4^+^ Foxp3^+^ tumor-infiltrating MAIT cells compared with Tregs upon 4 h of PMA/ionomycin stimulation (right); n = 6. Data are mean with SD from 2 experiments; two-tailed paired t test. See also Figure S2.

From the previous CyTOF analysis, the UMAP plot revealed a distinct cluster of CD4^+^ MAIT cells, some of which surprisingly co-expressed CTLA-4 and Foxp3 (Figure 2A). As shown with biaxial plots, we identified a population of CD4^+^ Foxp3^+^ tumor-infiltrating MAIT cells and validated them by flow cytometry on additional CRC tumor infiltrate samples (Figures 2B and S2A). No Foxp3^+^ MAIT cells were detected in the PBMCs of these patients, and only a few were observed in adjacent non-tumor tissue samples (Figure 2C). The frequency of CD4^+^ Foxp3^+^ MAIT cells in the tumors was heterogeneous and varied from 1% to 14% of total MAIT cells (Figure 2C). We found high expression of Helios, CTLA-4, and CD25 along with low expression of CD127 in these cells, similar to conventional Treg cells and in contrast to Foxp3^–^ MAIT cells (Figure 2D). These observations were confirmed by our scRNA-seq data, where few tumor-infiltrating MAIT cells expressing FOXP3 and co-expressing Treg-related surface markers were detected (Figure S2B). Although an association was observed between frequencies of CD4^+^ Foxp3^+^ MAIT cells and CD4^+^ MAIT cells, only a weak correlation was found between these cells and classical Tregs (Figures 2E and S2C). Functional analyses revealed an ability of CD4^+^ Foxp3^+^ MAIT cells to produce the pro-inflammatory cytokine tumor necrosis factor alpha (TNF-α), in contrast to Treg cells (Figure 2F). This result, supported by our scRNA-seq data, suggested that FoxP3 expression by tumor-infiltrating CD4^+^ MAIT cells was an indicator of activation rather than a marker of an immunosuppressive or regulatory subset (Figure S2D).^21^

### Enrichment of CD39 Expression on Tumor-Infiltrating MAIT Cells

**Figure 3:**
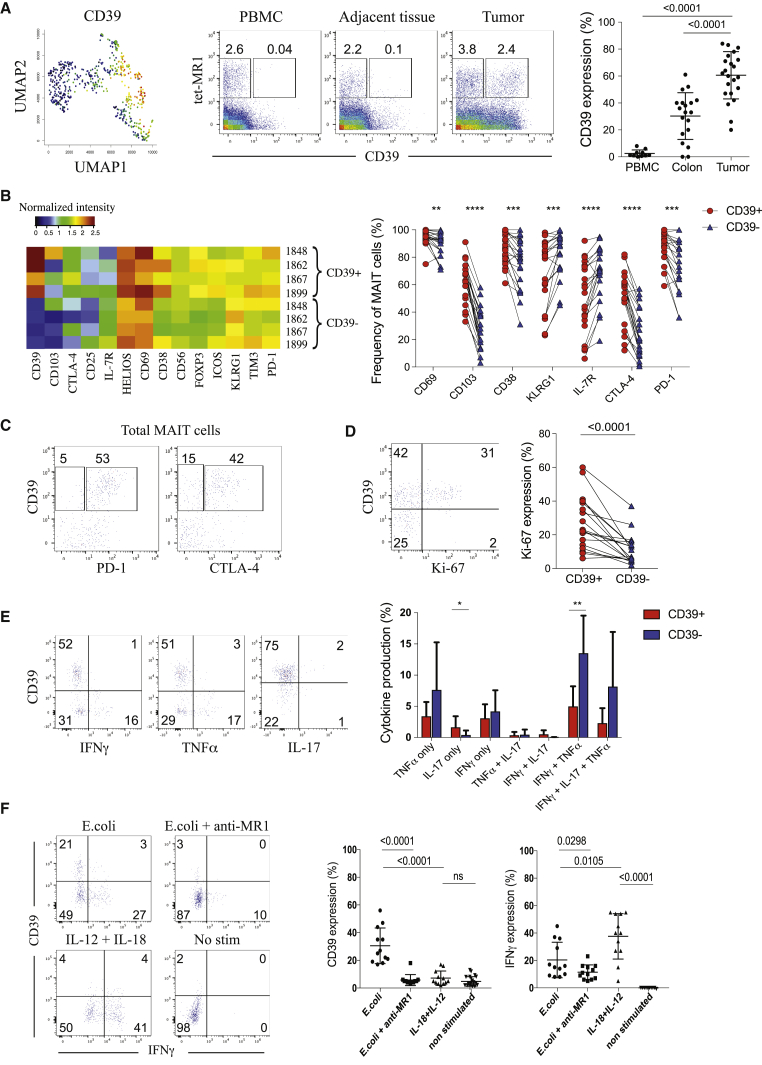
High Enrichment of CD39 Expression on Tumor-Infiltrating MAIT Cells (A) Left: CD39 expression shown by UMAP plot. Center: representative staining of CD39 expression on MAIT cells from PBMC, adjacent tissue, and tumor of CRC, gated on CD45^+^ live, DNA^+^, CD14^–^ CD16^–^ CD3^+^ T cells. Right: frequencies of CD39^+^ MAIT cells in different compartments. PBMC = 10, colon = 19, tumor = 24. Data are mean with SD from at least 7 experiments. Mann-Whitney U test. (B). Expression intensities of selected markers on CD39^+^ and CD39^–^ MAIT cells in 4 different tumors (left). The numbers are patient IDs. Also shown is analysis of selected markers expression between the CD39^+^ and CD39^–^ subset; n = 22 (right). Data are from at least 7 experiments. Two-tailed paired t test. (C) CD39 co-expression with PD-1 or CTLA-4 gated on total MAIT cells from one representative tumor sample. (D) Representative staining of CD39 and Ki-67 expression on tumor-infiltrating MAIT cells (left) and Ki67 expression on CD39^+^ and CD39^–^ tumor-infiltrating MAIT cells (right); n = 18. Data are from at least 3 experiments. Two-tailed paired t test. (E) Representative example of IFNγ, TNF-α, IL-17, and CD39 expression on tumor-infiltrating MAIT cells upon 4 h of PMA/ionomycin stimulation (left) and polyfunctionality profile of CD39^+^ and CD39^–^ tumor-infiltrating MAIT cells (right); n = 8. Data are mean with SD from 2 experiments. Two-tailed paired t test. (F) Flow cytometry data of CD39 and IFNγ expression after different stimulation conditions, assessed on CD3^+^ tet-MR1^+^ CD161^+^ cells; n = 12. Data are mean with SD from at least 3 experiments. Two-tailed paired t test. See also Figure S3.

From our CyTOF analysis and supported by our scRNA-seq data, we also observed enrichment of CD39 expression on tumor-infiltrating MAIT cells (Figures 1D and 3A). CD39 was expressed at lower frequencies in the adjacent tissue and was almost absent in PBMCs (Figure 3A). In line with a previous report by our group,^22^ we also observed a high frequency of CD39^+^ CD8^+^ tumor-infiltrating lymphocytes (TILs) in CRC (tet-MR1-negative T cells; Figure 3A). Although CD39^+^ MAIT cells strongly expressed the tissue-resident markers CD103 and CD69, KLRG1 and interleukin-7R (IL-7R) expression was reduced on these cells compared with the CD39^–^ population (Figures 3B and S3A). The inhibitory receptors PD-1 and CTLA-4 displayed elevated expression on the CD39^+^ subset (Figures 3B and 3C), supporting the previous scRNA-seq data, which identified higher expression of genes associated with exhaustion on tumor-infiltrating CD39^+^ MAIT cells, such as CTLA4 or HAVCR2 (Figure 1D). As we compared the phenotypic differences between MAIT cells from adjacent tissue and tumors, we also validated previous work reporting activation and tissue residency signatures for MAIT cells in human tissues (Figure S3B).^23^^,^^24^ We then explored the functional characteristics of CD39^+^ tumor-infiltrating MAIT cells. We observed that CD39^+^ MAIT cells expressed less active caspase than the CD39^–^ subset, suggesting protection against cell death (Figure S3C). Higher expression of Ki-67 was found in CD39^+^ MAIT cells compared with their CD39^–^ counterparts, indicating a higher proliferation rate (Figure 3D). We also evaluated the capability of these cells to produce cytokines, and our results indicated a lower polyfunctionality profile for CD39^+^ MAIT cells (Figure 3E). In this regard, no impairment in expression of Granzyme A or B or perforin was observed for CD39^+^ MAIT cells (Figure S3D). These cells showed higher expression of T-bet, PLZF, and Helios compared with the CD39^–^ subset (Figure S3E). No clear association between non-MAIT CD8^+^ CD39^+^ TILs and CD39^+^ MAIT cells was found, but a positive correlation was observed between the CD4^+^ Foxp3^+^ and CD4^+^ CD39^+^ MAIT subsets (Figure S3F). Previous studies reported CD39 to be expressed under chronic TCR stimulation on conventional T cells^25^ and on tumor-specific CD8^+^ T cells compared with cancer-unrelated CD8^+^ T cells.^22^^,^^26^^,^^27^ Because our results showed that tumor-infiltrating CD39^+^ MAIT cells were associated with an exhausted profile and a lower apoptosis and higher proliferation rate, they could be undergoing antigen exposure and chronically activated through their TCR as well. Using *E. coli* described to specifically stimulate MAIT cells in a TCR-driven manner,^28^ we performed *in vitro* assays and found that CD39 expression was induced after *E. coli* stimulation (Figure 3F). When stimulation was blocked with an anti-MR1 antibody, CD39 expression was strongly reduced, indicating TCR-dependent induction on peripheral MAIT cells. Interestingly, most CD39^+^ MAIT cells were found to be negative for IFNγ expression, suggesting a dichotomy between TCR-dependent CD39 induction and cytokine production (Figure 3F). Although cytokine stimulation (IL-18 and IL-12) induced IFNγ production by MAIT cells, it failed to induce any expression of CD39, arguing against non-TCR-driven cytokine signaling (Figure 3F).

### TCR-Dependent MAIT Cell Activation Is Associated with Tumor Infiltration by Bacteria

**Figure 4:**
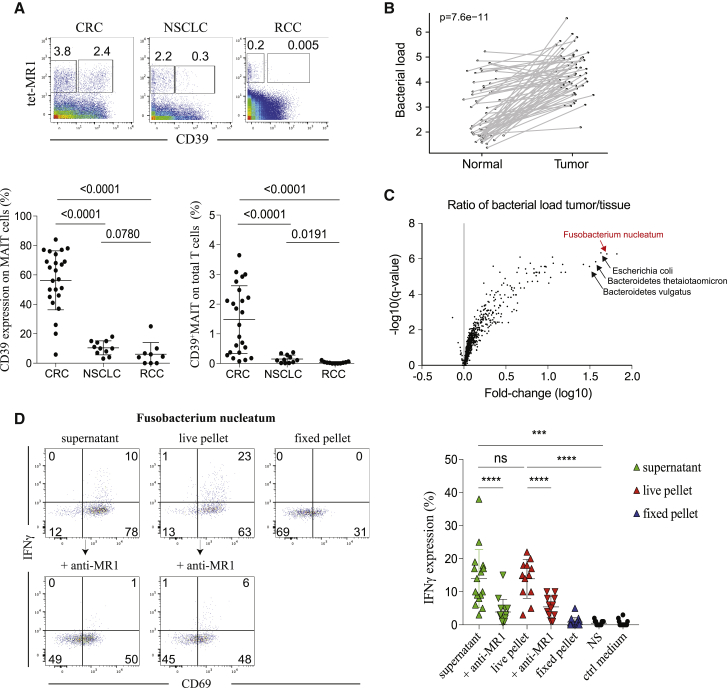
TCR-Dependent MAIT Cell Activation Is Associated with Tumor Infiltration by Bacteria (A) Representative staining of CD39 expression plotted against tet-MR1 in CRC, NSCLC and RCC tumor samples, gated on total T cells (top panel). Frequencies of CD39 expression on total MAIT cells and CD39^+^MAIT frequencies on total T cells (bottom panel). CRC = 24, NSCLC = 11, RCC = 9. Data are mean with SD from at least 10 experiments. Mann-Whitney U test. (B) Relative bacterial load (per sample) summed across all species in adjacent tissue versus corresponding CRC tumors. n = 61, two-tailed paired t test. (C) Ratio of of bacterial load in tumor versus tissue expressed in fold change(log10) versus p value expressed in -log10(q value). Bacterial species n = 1479. (D) Flow cytometry data of IFNγ production by MAIT cells stimulated under different conditions (STAR Methods); n = 12–16. Data are mean with SD from 6 experiments. Two-tailed paired t test. See also Figures S4 and S5.

Based on these results, we hypothesized that tumor-infiltrating MAIT cell phenotype and function could be shaped by tumor-invasive and adherent bacteria via production of MAIT cell-agonistic metabolites. As described above, we observed differences in the extent to which MAIT cells were infiltrating CRC versus NSCLC or RCC. Based on the consensus that the microbial biomass from the colon is much higher than those from the lungs and kidneys,^29^, ^30^, ^31^, ^32^ we expected that colon tumors would be most infiltrated by bacteria, followed distantly by the lungs and then the kidneys. In light of this, we particularly found much higher CD39 expression on tumor-infiltrating MAIT cells from CRC (Figure 4A). In PBMC, we did not see significant changes in the frequency of CD39^+^ MAIT cells across the three types of cancer (Figure S4A), supporting the idea that acquisition of CD39 expression was tumor specific. Of note, we observed a fairly conserved expression profile of tumor-infiltrating MAIT cells across the three tumor types, including high expression of tissue-resident and inhibitory markers, highlighting the specificity of CD39 expression on CRC tumor-infiltrating MAIT cells as being antigen specific (data not shown). To assess relationships between tumor infiltration by bacteria and the profiles of MAIT cells within CRC tumors, we analyzed data from whole-genome sequencing of tumor and paired adjacent tissue samples. Bacterial composition was determined using the metagenomic sequence classification software Kraken.^33^ The bacterial load was higher in the tumor compared with the paired adjacent tissue (Figure 4B), which fits our observation of elevated CD39 expression by MAIT cells in tumors compared with healthy adjacent tissue (Figures 3A and S3B). Further analysis of the bacterial load in tumor versus adjacent tissue highlighted a few bacterial strains that were particularly enriched in these tumors, including *Escherichia coli* (Figure 4C). Because most bacterial species were barely detected, we focused our analysis on species with the highest abundance (more than 100 log_10_ counts across all tumor samples) and generated a biclustering graph of relative bacterial loads across tumor samples only. Using hierarchical clustering, we observed that the bacterial load across tumors was heterogeneous and that the abundances of bacteria from the same phylum were overall correlated (Figure S4B). From a subset of tumors for which MAIT cell phenotyping and whole-genome sequencing data were available (n = 26), we found that the frequency of CD39^+^ MAIT but not CD39^+^CD8^+^ cells in tumors was fairly correlated with the bacterial load obtained from the same patient (Figure S4C). As an additional analysis to support our hypothesis of CD39 expression on MAIT cells associated with tumor bacterial infiltration, we compared the percentage of CD39^+^ MAIT cells in highly bacterially infiltrated tumors versus the ones in the middle of the biclustering graph displaying a low bacterial load (Figure S4B). Despite the low number of samples with data on CD39 expression, we observed that, overall, tumors with low bacterial infiltration showed lower levels of CD39 expression on MAIT cells (Figure S4D).

We next hypothesized that certain gut bacterial strains associated with CRC could modulate MAIT cell function. From the bacteria identified in Figures 4C and S4B, we selected few strains of *Bacteroides* and *Fusobacterium* that were present in tumors with a high load for *in vitro* stimulation assays. In particular, *Fusobacterium nucleatum* is widely reported to correlate with CRC pathogenesis.^34^, ^35^, ^36^, ^37^, ^38^ We co-cultured *Bacteroides thetaiotaomicron*, *Bacteroides vulgatus*, or *Fusobacterium nucleatum* with HD PBMCs and observed that only *F. nucleatum* was able to activate MAIT cell and stimulate cytokine production (Figure S4E). Compared with different co-culture conditions, both live bacteria and culture supernatants from *F. nucleatum* were able to activate MAIT cells (Figure 4D). Anti-MR1 blocking assays dramatically reduced IFNγ production, arguing that MAIT cell activation by *F. nucleatum* was mediated through a TCR-dependent signal (Figure 4D). These results implicate *F. nucleatum* as a potential bacterial modulator of MAIT cell response in CRC.

Because it would be important to establish the role of tumor-infiltrating MAIT cells in CRC, we explored some of the defined MAIT cell features in the context of patient clinical outcome. First, based on the consensus molecular classification (CMS) used in CRC,^39^^,^^40^ we found significant effects of CMS on bacterial composition, with the highest bacterial infiltration in the CMS3 subtype, suggesting a low immune and inflammatory signature (Figure S5A). We then considered expression of genes involved in tissue repair and wound healing functions for MAIT cells^41^^,^^42^ and found that CCL3, CSF1, and EGR1 were significantly more expressed in tumor-infiltrating MAIT cells versus PBMC, arguing for a role of tumor-infiltrating MAIT cells in promoting epithelial growth (Figure S5B). Despite a trend toward a higher frequency of MAIT cells in later-stage CRC patients, our analysis related to different clinical parameters was not conclusive because of inadequate statistical power (Figures S5C–S5E). Finally, we explored the role of riboflavin biosynthesis gene expression in CRC prognosis and found that diaminohydroxyphosphoribosylaminopyrimidine deaminase (EC:3.5.4.26), also known as PyrD, was enriched in the CRC metagenome (Figure S5F; data from Minot and Willis^43^). Knowing that PyrD is necessary for MAIT cell development and activation,^44^ this result might support the importance of MAIT cell infiltration and activation in CRC.

## Discussion

Our study aimed to decipher the role of MAIT cells in human cancer.^45^, ^46^, ^47^ Although we observed a presence of MAIT cells in different cancer types, only MAIT cells from CRC were characterized by high expression of CD39, which we showed to be induced in a TCR-dependent manner. In line with our findings, a recent work associated chronic TCR stimulation and decreased responsiveness of gut MAIT cells with upregulation of PD-1 and CD39 expression.^48^ We also identified a Foxp3^+^ MAIT cell subset in tumors with a surface marker expression pattern similar to conventional Treg cells. The finding that they produced TNF-α and, to a lesser extent, IL-17 suggests pro-inflammatory potential. MAIT cells may possess an IL-17-producing subset derived from the periphery upon TCR activation in the presence of a pro-inflammatory milieu, as seen previously for non-MAIT CD4^+^ FOXP3^+^ T cells.^49^ With this in mind, the higher IL-17 production observed by flow cytometry and expression of the inflammatory genes RSG1, CCL3, and CCL4 revealed by our scRNA-seq data could support a pro-tumorigenic role of CD39 expression in tumor-infiltrating MAIT cells in CRC.

We showed the capacity of *Fusobacterium nucleatum* to activate MAIT cells in a TCR-dependent manner. The reported high presence of this species in CRC versus NSCLC or RCC^50^ strengthens the idea that TCR-mediated activation of MAIT cells depends on tumor microbiome composition and/or diversity. Although we believe in the potential role of *Fusobacterium nucleatum* in modulating the MAIT cell response in tumors, it is also conceivable that the latter could result from a synergistic response, given the substantial bacterial infiltration in CRC, and/or could be helped by additional mediators from the tumor microenvironment. Extended studies with tumor cultures or implementing colonic organoid systems could allow validation and further exploration of MAIT cells. This will help us to evaluate the tumor specificity of this response and develop new tools for targeted cancer therapies.

Following a previous study of our group focusing on CD39^+^ CD8^+^ TILs,^22^ we aimed to assess MAIT cells as antigen-specific versus innate-like bystanders in this context. Our results indicate that, from different aspects, CD39 expression on MAIT cells can be considered a marker of TCR-mediated antigen recognition and is associated with hallmarks of T cell exhaustion as in conventional CD8^+^ T cells. So far, two reports have associated high infiltration of MAIT cells in tumors with a bad prognosis;^51^^,^^52^ here we consider that several MAIT cell features possibly contribute to CRC development, including TCR-driven CD39 expression, tumor bacterial antigen recognition, pro-inflammatory signals, and riboflavin gene expression. Understanding what makes MAIT cells detrimental in the context of cancer is crucial, and unveiling their association with the tumor microbiome strongly supports the interest in manipulating the gut microbiome to reshape their response. Consequently, this study opens an avenue for modulating tumor immunity by targeting MAIT cells directly or indirectly through alterations in the microbiome that would be designed to specifically modulate MAIT cell activity.

### Limitations of Study

First, because of the limited number of Foxp3^+^ MAIT cells detected in the tumor infiltrate samples, additional studies will be needed to more properly assess the function of these cells. Second, because *in vitro* conditions do not fully mimic the tumor microenvironment and because MAIT cell ligands are highly unstable products,^11^ it would be important to evaluate how bacterial dose- and/or timing-dependent stimulations can affect MAIT cell activation as well as differential expression of pertinent genes, differences in the concentration of the metabolite produced by the bacteria, etc. Last, and mostly because of inadequate statistical power, this study indicates but falls short of fully demonstrating the effect of CD39^+^MAIT cells on the tumor microenvironment.

## STAR★Methods

### Key Resources Table

**Table:** 

REAGENT or RESOURCE	SOURCE	IDENTIFIER
**Antibodies**		

89 - CD45 (clone HI30)	Fluidigm	Cat# 3089003, RRID:AB_2661851
112/114 - CD14 (clone TuK4)	Thermofisher	Cat# MHCD1400, RRID:AB_10371749
115 - CD57 (clone HCD57)	Biolegend	N/A
140 - CD28 (clone CD28.2)	Biolegend	Cat# 302902, RRID:AB_314304
140 - CD4 (clone RPA-T4)	Biolegend	Cat# 300502, RRID:AB_314070
141 - CD56 (clone NCAM16.2)	BD	Cat# 559043, RRID:AB_397180
142 - HLA-DR (clone L243)	Biolegend	Cat# 307602, RRID:AB_314680
143 - CD3 (clone UCHT1)	Biolegend	Cat# 300402, RRID:AB_314056
144- IL18R (clone H44)	Biolegend	Cat# 313804, RRID:AB_345312
145- CD69 (clone FN50)	Biolegend	Cat# 310902, RRID:AB_314837
146 - CD8 (clone RPA-T8)	Biolegend	Cat# 301002, RRID:AB_314120
147 - CD45RA (clone HI100)	Biolegend	Cat# 304102, RRID:AB_314406
147 - CD150 (clone A12)	Biolegend	Cat# 306302, RRID:AB_314590
147 - CD4 (clone RPA-T4)	Biolegend	Cat# 300502, RRID:AB_314070
148 - CD45RO (clone UCHL1)	Biolegend	Cat# 304202, RRID:AB_314418
149 - CCR6 (clone G034E3)	Biolegend	Cat# 353402, RRID:AB_10918625
150 - CD103 (clone B-Ly7)	Thermofisher	Cat# 14-1038-80, RRID:AB_467411
151 - KLRG1 (clone 13F12F2)	Thermofisher	Cat# 16-9488-85, RRID:AB_2637116
152 - 2B4 (clone C1.7)	Biolegend	Cat# 329502, RRID:AB_1279194
152 - ICOS (clone C398.4A)	Biolegend	Cat# 313502, RRID:AB_416326
153 - CD25 (clone M-A251)	Biolegend	Cat# 356102, RRID:AB_2561752
154 - CCR4 (clone MAB1567_100)	R&D	Cat# MAB1567, RRID:AB_2074395
154 - CD186 (clone K041E5)	Biolegend	Cat# 356002, RRID:AB_2561738
155 - Tbet (clone eBio4B10)	Thermofisher	Cat# 14-5825-82, RRID:AB_763634
156 - GATA3 (clone TWAJ)	Thermofisher	Cat# 14-9966-82, RRID:AB_1210519
157 - TIM3 (clone F38-2E2)	Biolegend	Cat# 345002, RRID:AB_2116574
158 - CD38 (clone HIT2)	Biolegend	Cat# 303502, RRID:AB_314354
159 - CD161 (clone HP-3G10)	Biolegend	Cat# 339902, RRID:AB_1501090
160 - PD-1 (clone eBioJ105)	Thermofisher	Cat# 14-2799-80, RRID:AB_763476
161 - PLZF (clone MAB2944)	R&D	Cat# MAB2944, RRID:AB_10718564
162 - CX3CR1 (clone K0124E1)	Biolegend	Cat# 355702, RRID:AB_2561726
162 - CXCR3 (clone 49801)	R&D	Cat# MAB160, RRID:AB_2086754
162 - TNFa (clone MAB11)	Biolegend	Cat# 502902, RRID:AB_315254
163 - il7r (clone AO19D5)	Biolegend	Cat# 351302, RRID:AB_10718513
164 - VD1 FITC (clone REA173)	Miltenyi	Cat# 130-100-534, RRID:AB_2653951
165 - Va7.2 (clone 3C10)	Biolegend	Cat# 351702, RRID:AB_10900258
166 - CCR9 (clone L053E8)	Biolegend	Cat# 358902, RRID:AB_2562298
166 - NKP46 (clone 195314)	R&D	Cat# MAB1850, RRID:AB_2149153
166 - CCR5 (clone HEK/1/85a)	Abcam	N/A
166 - CD107a (clone H4A3)	Biolegend	Cat# 328602, RRID:AB_1134259
167 - gd PE (clone B1)	Biolegend	Cat# 331210, RRID:AB_1089218
168 - CCR7 (clone 150503)	R&D	Cat# MAB197, RRID:AB_2072803
168 - il17a (clone BL168)	Biolegend	Cat# 512302, RRID:AB_961399
169 - CD85J (clone GHI/75)	Biolegend	Cat# 333702, RRID:AB_1089089
169 - HELIOS (clone 22F6)	Biolegend	Cat# 137202, RRID:AB_10900638
171 - VD2 (clone B6)	Biolegend	Cat# 331402, RRID:AB_1089226
172 - VG9 (clone B3)	Biolegend	Cat# 331301, RRID:AB_1089235
173 - vd1 apc (clone REA173)	Miltenyi	Cat# 130-100-519, RRID:AB_265395
174 - CD160 (clone 688327)	R&D	Cat# MAB6700, RRID:AB_10891689
174 - TIGIT (clone MAB7898)	R&D	Cat#MAB7898
174 - CTLA4 (clone BNI3)	BD	Cat# 555851, RRID:AB_396174
174 - granzyme B (clone CLB-GB11)	Thermofisher	Cat# MA1-10338, RRID:AB_11154492
175 FOXP3 BIOTIN (clone PCH101)	Thermofisher	Cat# 13-4776-82, RRID:AB_763539
175 perforin (clone B-D48)	Abcam	Cat# ab47225, RRID:AB_2169084
176 - CD19 (clone HIB19)	Biolegend	Cat# 302202, RRID:AB_314232
176 - CD39 (clone A1)	Biolegend	Cat# 328202, RRID:AB_940438
176 - CD26 (clone BA5b)	Biolegend	Cat# 302702, RRID:AB_314286
209 - CD16 (clone 3G8)	Fluidigm	Cat# 3209002B, RRID:AB_2756431
CD161 (clone HP-3G10)	Biolegend	Cat# 339916, RRID:AB_2563607
CD3 (clone OKT3)	Biolegend	Cat# 317324, RRID:AB_2563352
CD45 (clone HI30)	Biolegend	Cat# 304042, RRID:AB_2562106
CD39 (clone A1)	Biolegend	Cat# 328212, RRID:AB_2099950
CD4 (clone RPA-T4)	Biolegend	Cat# 300546, RRID:AB_2563314
CD8 (RPA-T8)	Biolegend	Cat# 301033, RRID:AB_1595443
IFNg (clone 4S.B3)	Thermofisher	Cat# 11-7319-82, RRID:AB_465415
TNFa (clone MAb11)	BD	Cat# 560679, RRID:AB_1727579
IL17 (clone N49-653)	BD	Cat# 563745, RRID:AB_2738401
Va7.2 (clone 3C10)	Biolegend	Cat# 351716, RRID:AB_2563864
7-AAD	Thermofisher	Cat#A1310
Annexin V	Biolegend	Cat#640906
PI	Thermofisher	Cat#P1304MP
FLICA	Thermofisher	Cat# V35118
APC streptavidin	Biolegend	Cat#405207
PE streptavidin	Biolegend	Cat#405204

**Bacterial and Virus Strains**		

Bacteriodes thetaiotamicron	DSM	2079 (VPI 5482)
Bacteroides vulgatus	ATCC	8482
Fusobacterium nucleatum	ATCC	25586
*Escherichia coli*	Invitrogen	18265017

**Biological Samples**		

CRC tumor, healthy tissue and blood samples	Singapore	N/A
NSCLC tumor, healthy tissue and blodd samples	Singapore	N/A
RCC tumor, healthy tissue and blood samples	North-West BiosTrust	N/A

**Chemicals, Peptides, and Recombinant Proteins**		

MR1 tetramer	NIH tetramer core	N/A
CD1d tetramer	NIH tetramer core	N/A

**Critical Commercial Assays**		

Rhapsody AbSeq reagent pack	BD Biosciences	Cat#633771
Rhapsody Human T cell expression panel	BD Biosciences	Cat#633751
Human Single cell multiplexing kit	BD Biosciences	Cat#633781

**Deposited Data**		

Sc-RNaseq/AbSeq data	https://www.ncbi.nlm.nih.gov/geo/	GEO: GSE151842

**Experimental Models: Cell Lines**		

THP-1	ATCC	TIB-202

**Software and Algorithms**		

R Studio and R environment	The R project for Statistical Computing	https://rstudio.com/ and https://cran.r-project.org/
Seven Bridges (pre-processing of Rhapsody FASTQ files)	BD Biosciences	https://www.sevenbridges.com

### Resource Availability

#### Lead Contact

Further information and requests for resources and reagents should be directed to and will be fulfilled by the Lead Contact, Evan W. Newell (enewell@fredhutch.org).

#### Materials Availability

This study did not generate new unique reagents.

#### Data and Code Availability

The accession number for the scRNAseq data discussed in this study is GSE15184 at NCBI's Gene Expression Omnibus.

### Experimental Model and Subject Details

#### Human samples

Blood, tumors and adjacent tissues samples were collected from patients with Colorectal Cancer (CRC), Non-Small Cell Lung Cancer (NSCLC) or Renal cell carcinoma (RCC). The use of human tissues was approved by the appropriate institutional research boards, A∗STAR and the Singapore Immunology Network, Singapore. The RCC samples were provided by Northwest Biotrust, under a NWBiospecimens protocol, Seattle. NW BioTrust, a core service for patient consenting, and NWBioSpecimen, a core service for procurement and annotation of research biospecimens, are supported by National Cancer Institute grant P30 CA015704 (G. Gilliland, principal investigator [PI]), Institute of Translational Health Sciences grant UL1 TR000423 (M. Disis, PI), the University of Washington School of Medicine and Department of Pathology, and Fred Hutchinson Cancer Research Center. The analysis was performed according to the IRB file/approval number NHS #6007-1061. No age or gender information is available. Informed written consent was obtained from each subject or each subject’s guardian.

#### Bacterial strains

DH5α *E. coli* was aerobically cultured overnight in a shaking incubator at 37°C in Luria-Bertani broth. *Bacteroides thetaiotaomicron* and *Bacteroides vulgatus* were anaerobically cultured for 18-24 hours in modified gut microbiota media as previously described.^53^ *Fusobacterium nucleatum* was anaerobically cultured for 18-24 hours in fastidious anaerobic broth as previously described.^54^ Bacterial growth of all strains was estimated by measuring optical densities at 600 nm (OD_600_).

### Method Details

#### Cell isolation

Samples were prepared as previously described.^22^ Briefly, tissues were mechanically dissociated into small pieces and incubated at 37°C for 10-20min in DMEM + collagenase IV (1mg/ml) + DNase (10ug/ml). Digestion was stopped by addition of RPMI+5% FBS. Dissociated tissues were filtered and washed twice before cryopreservation in freezing medium (90% FBS and 10% DMSO). PBMCs from patients and healthy donors were isolated from peripheral blood samples by Ficoll separation and washed twice with PBS before cryopreservation.

#### CyTOF staining

Purified antibodies lacking carrier proteins were purchased according to the provided in the Key Resource Table. Antibody conjugation was performed according to the protocol provided by Fluidigm. Streptavidin was labeled as previously described.^55^ MR1- and CD1d-tetramers are provided by the NIH Tetramer Core Facility and were tetramerized as stated with our metal-labeled streptavidins. Frozen samples were thawed and washed twice in thawing medium (RPMI, 10% FBS, 10 μg/ml DNase). Cells were first enriched for CD45 using purified anti-CD45 antibody followed by anti-mouse IgG microbeads, and positively selected using MACS LS columns. Cells were then stained for cisplatin (5 μM) in PBS for 5 min on ice, washed and incubated for 1h at room temperature with MR1-tetramer. Cells were then incubated with primary and secondary surface antibodies cocktails for 20min each on ice, fixed and permeabilized using Foxp3 transcription factor staining buffer set (eBioscience) and stained for primary and secondary intranuclear antibodies for 30min each on ice. Cells were then washed three times and fixed overnight in PFA 2%.

The next day, the cells were washed twice before barcoding with in-house barcodes in PBS as previously described for 30 min at 4°C,^56^ washed and stained for DNA for 15 min at room temperature (Cell-ID intercalator-Ir, Fluidigm). Cells were lastly washed three times with dH2O, counted and run on CyTOF at a ratio of 0.5M cells/ml in dH2O.

#### *in vitro* MAIT cell stimulation

MAIT cells were stimulated for 16h with either IL-12+IL-18 (R&D) at 50ng/ml or different strains of bacteria as described above. For *E. coli*, bacteria were fixed using 2% PFA and washed extensively in PBS. For *B. thetaiotaomicron, B. vulgatus*, and *F. nucleatum,* either culture supernatant, live or fixed cell pellets were used. Bacterial cells were pelleted by centrifugation (15,000xg for 5 minutes), and any remaining bacterial cells were removed from the supernatant through syringe-filtration (0.2 μM). Next, 1M of growing ThP-1 cells (ATCC® TIB-202) were incubated with 25M of either E.coli or other bacteria in ThP-1 culture medium (RPMI-1640 + 0.05mM of 2-mercaptoethanol and 10% of FBS). 4-5h later, bacteria were washed away and 2M of PBMCs were added to the culture and stimulated for 16h. BFA (eBioscience) was added for the last 5 hours to stop the stimulation. For MR1-blocking assay, purified anti-MR1 antibody (clone 26.5, Biolegend) was added to the culture before the PBMCs and incubated for 20 min at 4°C. The activation of MAIT cells was then measured through IFNγ, TNFα or IL-17a expression by Flow cytometry.

#### FACS analysis

Frozen samples were thawed as mentioned above and surface stained for FACS using antibodies listed in the Key Resource Table. For Foxp3 staining, cells were fixed and permeabilized using Foxp3 transcription factor staining buffer set, incubated with biotin anti-FoxP3 and then APC streptavidin for 30 min each at 4°C. For apoptosis assay, cells were stained using Vybrant FAM Poly Caspases Assay kit (ThermoFisher) and according to manufacturer’s instructions. Samples were run on BD FACSCelesta or LSRFortessa and analyzed using FlowJo.

### Quantification and Statistical Analysis

#### CyTOF data analysis and UMAP

After mass cytometry (CyTOF) acquisition, any zero values were randomized using a uniform distribution of values between 0 and −1 using R. The signal of each parameter was normalized based on EQ beads (Fluidigm) as described previously.^57^ Cells were manually debarcoded using FlowJo. Samples were then used for UMAP analysis similar to that previously described using customized R scripts based on the ‘flowCore’ and ‘uwot’ R packages.^19^ In R, all data were transformed using the logicleTransform function (flowCore package) using parameters: *w* = 0.25, *t* = 16409, *m* = 4.5, *a* = 0 to roughly match scaling historically used in FlowJo. For heatmaps, median intensity corresponds to a logical data scale using formula previously described.^58^ The colors in the heatmap represent the measured means intensity value of a given marker in a given sample. A seven-color scale is used with black–blue indicating low expression values, green–yellow indicating intermediately expressed markers, and orange-red representing highly expressed markers.

#### mRNA sequencing and Gene enrichment analysis

Gene expression profiles of tumor-infiltrating MAIT cells were obtained from FACS-sorted MAIT cells using tet-MR1 and CD161. Paired-end RNA-Seq reads from Illumina HiSeq 4000 were counted based on the human GENCODE V27 gene annotations^59^ using Salmon^60^ to the gene level. The logarithmically transformed counts were filtered for IQR (Inter Quartile Range) > 0.5. The filtered genes counts were used in edgeR^61^ to identify the differentially expressed genes (DEG) on genes. Multiple testing correction was conducted using the method of Benjamini and Hochberg with adjusted P values < 0.05 deemed to be significant. All analyses were done in R v.3.3.3. We ran PCA using the prcomp function from the stats (v.3.4.2) R package, using the whole transcriptomic data. We used the HTSanalyzeR package (v.2.26.0) to run GSEA on gene collections from the Gene Ontology Biological Processes database, filtered for gene sets with at least 20 genes present in our dataset. For GSEA we used 1000 permutations to estimate *P*values and applied corrections for multiple tests using the Benjamini–Hochberg procedure.

#### BD AbSeq single-cell mRNA sequencing

Tumor-infiltrating MAIT cells from 3 CRC patients and control PBMC from 1 healthy donor were obtained by FACS sorting using tet-MR1. Cells were then incubated with BD AbSeq Ab-oligos following manufacturers’ instructions (BD Biosciences). Single cells were isolated using Single Cell Capture and cDNA synthesis with the BD Rhapsody Express Single-cell Analysis System. Parallel RNA and BD AbSeq sequencing libraries were generated using BD Rhapsody targeted mRNA and AbSeq amplification and BD Single-cell Multiplexing kits and protocol. Quality of final libraries was assessed using Agilent 2200 TapeStation with High Sensitivity D5000 ScreenTape, quantified using a Qubit Fluorometer (ThermoFisher), and carried through to sequencing with Novaseq S1 on Illumina sequencer. FASTQ files containing sequenced data were analyzed using the Seven Bridges platform provided by BD (See “BD Single Cell Genomics Bioinformatics Handbook – 54169 Rev. 6.0” for specific details).^20^ The output files containing proteins and genes expression for each cell were transformed using log2 with a pseudo count of 1 and analyzed for differentially expressed genes or proteins using the *FindMarkers* function of the Seurat (v2.3.4) R package^62^ using a Wilcoxon test (default) as differential expression method and an expression frequency threshold of 0.

#### DNA extraction, WGS and metagenomic analysis

DNA Sequencing was performed as previously reported.^63^ In brief, ten 5-um tissue sections were used to obtain tissue for whole genome sequencing (WGS). DNA-Seq libraries were prepared using the KAPA Hyper Prep Kit (Kapa Biosystems, Wilmington, MA) and sequenced on the Illumina HiSeq platform (Illumina, San Diego, CA). Raw sequencing reads were aligned to the human genome (hg19 assembly) using the BWA aligner.^64^ Reads that were not mapped to the human genome based on this strict criterion were assigned to bacterial species using the Kraken software^33^ with the MiniKraken database and default parameters.

#### Expression of riboflavin genes in CRC metagenome

Violin plot of the estimated coefficients in CRC versus healthy patient metagenome for different microbiome-encoded genes involved in riboflavin metabolism (grouped by their enzyme commission (EC) number). Analysis performed with data published in Minot et al., Microbiome, 2019.^43^ A positive number indicates that the gene is enriched in the CRC metagenome, whereas a negative number indicates that the gene is enriched in the healthy metagenome. The first plot is for all genes except the riboflavin metabolism genes. Fisher’s exact test was used for each riboflavin gene to assess for association with cancer compared to all other genes.

## Supplemental Information

Document S1. Figures S1–S5 and Data S1

Document S2. Article plus Supplemental Information
