# Supplementary material for: Human Tumor-Infiltrating MAIT Cells Display Hallmarks of Bacterial Antigen Recognition in Colorectal Cancer
Source: Cell Rep Med. 2020 Jun 23;1(3):100039. doi: 10.1016/j.xcrm.2020.100039 (PMC7659584; doi:10.1016/j.xcrm.2020.100039)
Supplement: Document S1. Figures S1–S5 and Data S1 [file mmc1.pdf]

Cell Reports Medicine, Volume *1*

Supplemental Information

Human Tumor-Infiltrating MAIT Cells Display Hallmarks of Bacterial Antigen Recognition in Colorectal Cancer

Shamin Li, Yannick Simoni, Etienne Becht, Chiew Yee Loh, Naisi Li, Daniel Lachance, Si-Lin Koo, Teck Por Lim, Emile Kwong Wei Tan, Ronnie Mathew, Andrew Nguyen, Justin Golovato, Julia D. Berkson, Martin Prlic, Bernett Lee, Samuel S. Minot, Niranjan Nagarajan, Neelendu Dey, Daniel S.W. Tan, Iain B. Tan, and Evan W. Newell

Supplementary Figure S1

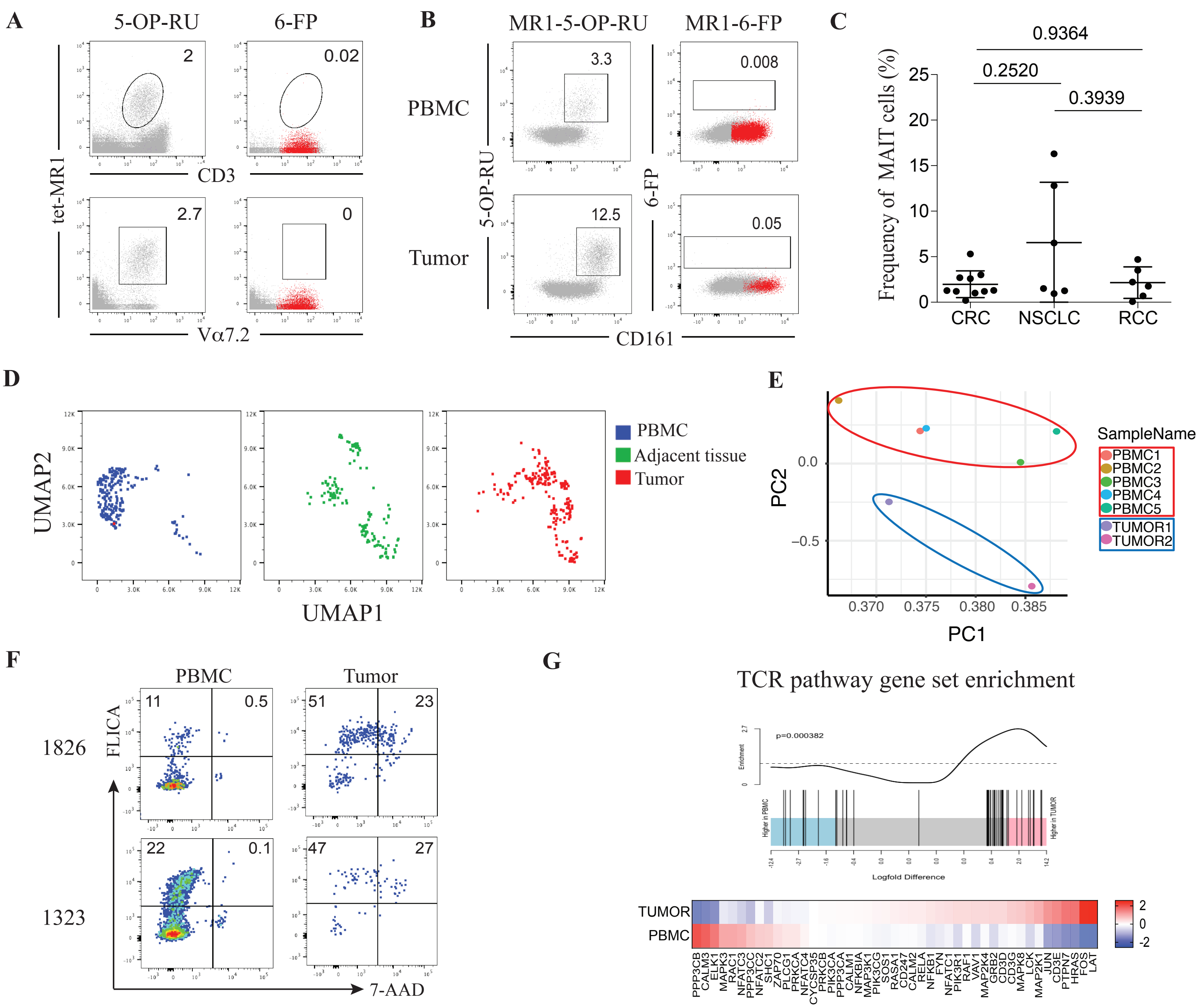

Supplementary figure S1: Tumor-infiltrating MAIT cells express more apoptotic signals and a higher TCR signaling, related to Figure 1. **A**. Representative staining of MAIT cells from HD PBMC by mass cytometry, as gated on MR1-tet loaded with 5-OP-RU or 6-FP (negative control), along with CD3 on total live $CD45^{+}CD14^{-}CD16^{-}$ cells (upper panel), with Vα7.2 on total $CD45^{+}CD14^{-}CD16^{-}CD3^{+}$T cells (lower panel). The MR1-6-FP^{+} cells are shown in purple on the left panel, the MR1-5-OP-RU^{+}CD161^{+} cells are shown in red on the right panel. **B.** Representative tet-MR1 staining (5-OP-RU and control 6-FP) gated on $CD45^{+}CD3^{+}$ T cells from healthy donor (HD) PBMC or CRC tumor by flow cytometry. The MR1-6-FP^{+} cells are shown in purple on the left panel, the MR1-5-OP-RU^{+}CD161^{+} cells are shown in red on the right panel. **C.** Frequency of total MAIT cells in PBMC of CRC (n=10), NSCLC (n=6), RCC (n=6) patients. Data are mean with S.D. from at least 7 experiments. Mann-Whitney U test. **D**. Individual UMAP plot analyzing MAIT cell phenotype from each compartment. **E**. PCA analysis of the transcriptomic profile of MAIT cells from tumors (blue circle) as compared to PBMCs (red circle). Tumors and PBMCs are paired for the samples 1 and 2. **F**. Representative staining showing MAIT cell apoptosis in two tumors and paired PBMCs, as assessed by FLICA (caspase activation) and 7-AAD staining. The numbers are patients ID. **G.** Gene Set Enrichment Analysis of MAIT cells in tumors as compared to the paired PBMCs, based on the genes from the TCR pathway (upper panel). Heatmap of genes that are differentially expressed in MAIT cells between tumors and PBMCs (lower panel). The colors indicate the expression intensities, from low (blue) to high (red).

Supplementary figure S2

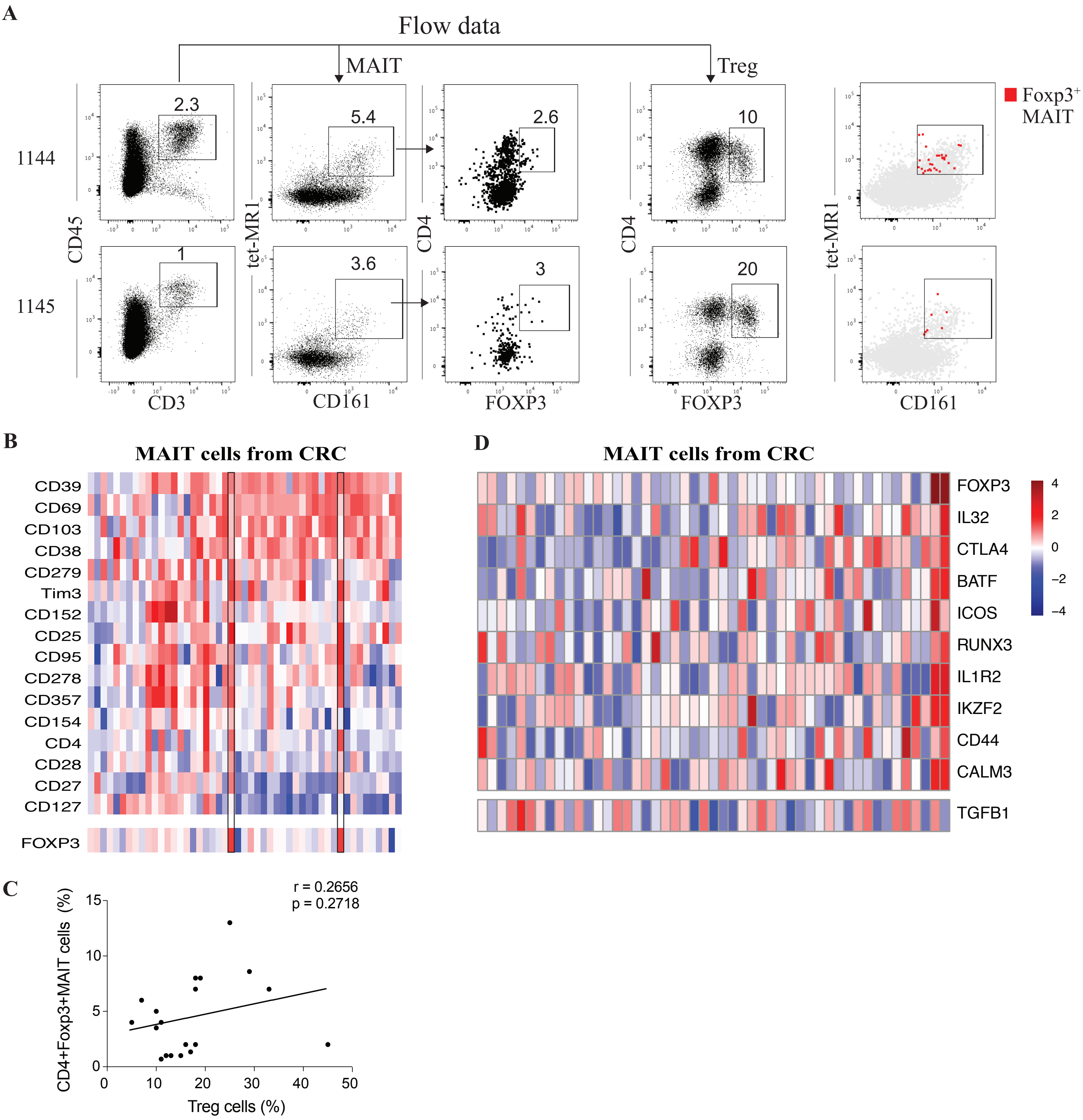

Supplementary figure S2: Tumor-infiltrating CD4$^+$Foxp3$^+$MAIT cells in CRC, related to Figure 2.

A. Representative flow cytometry staining of CD4$^+$Foxp3$^+$MAIT cells in two colorectal tumor samples, as compared with conventional Treg cells from the same tumor (left panel). Backgating of CD4$^+$Foxp3$^+$MAIT cells (red) on total MAIT cells on the same samples (right panel). **B.** Heatmap showing expression intensities of selected surface proteins vs. FOXP3 in MAIT cells from CRC tumor. Single-cell targeted mRNAsequencing combined with BD ABseq Rhapsody system on sorted MAIT cells (see methods). **C.** Correlation of Foxp3 expression on MAIT cells with Treg cells, n=20. **D.** Heatmap showing expression intensities of selected activation genes and TGFB1 vs. FOXP3 in tumor-infiltrating MAIT cells.

Supplementary figure S3

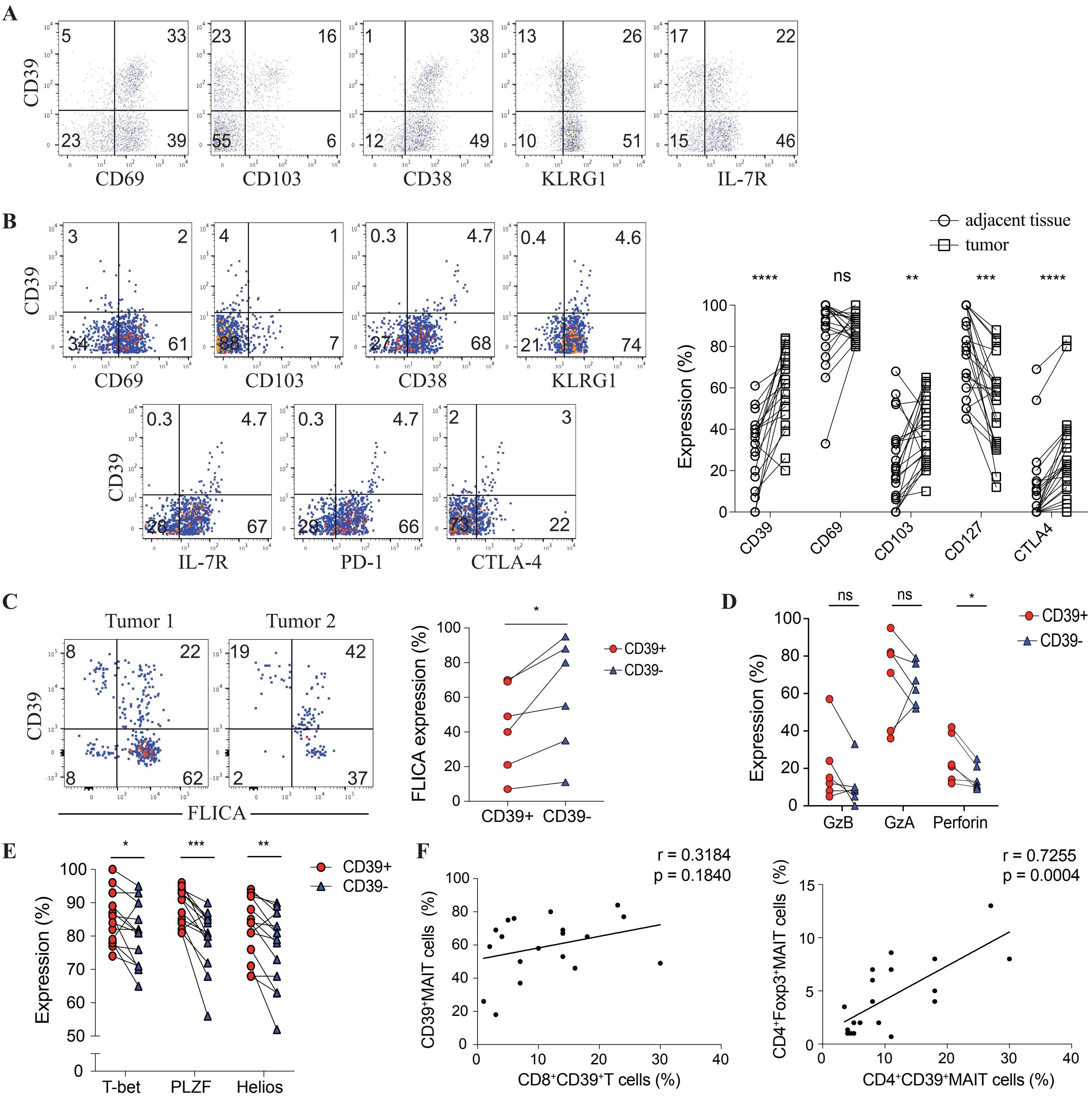

Supplementary figure S3: Tumor-infiltrating CD39^{+}MAIT cells in CRC, related to Figure 3.

A. Coexpression of selected markers with CD39 on tumor-infiltrating MAIT cells from one representative CRC patient. **B.** Coexpression of selected markers with CD39 on MAIT cells of adjacent colon tissue from one representative CRC patient (left panel). Comparison of selected markers expression between MAIT cells from adjacent tissue and tumor (right panel), n=19-22, Two-tailed paired t-test. **C.** Expression of FLICA (caspase activation) and CD39 on tumor-infiltrating MAIT cells. n=6. Data are from 2 experiments. Two-tailed paired t-test. **D.** Expression of Granyme B, Granzyme A and perforin on CD39^{+} vs. CD39^{-} tumor-infiltrating MAIT cells upon PMA/Iono stimulation. n=6. Data are from 2 experiments. Two-tailed paired t-test. **E.** Expression of T-bet, PLZF and Helios on CD39^{+} vs. CD39^{-} tumor-infiltrating MAIT cells, n=14. Data are from at least 3 experiments. Two-tailed paired t-test. **F.** Correlation of CD39 expression on MAIT cells with CD8^{+}CD39^{+}T cell frequencies, CD4^{+}Foxp3^{+}MAIT cells with CD4^{+}CD39^{+}MAIT cell frequencies, n=20.

Supplementary Figure S4

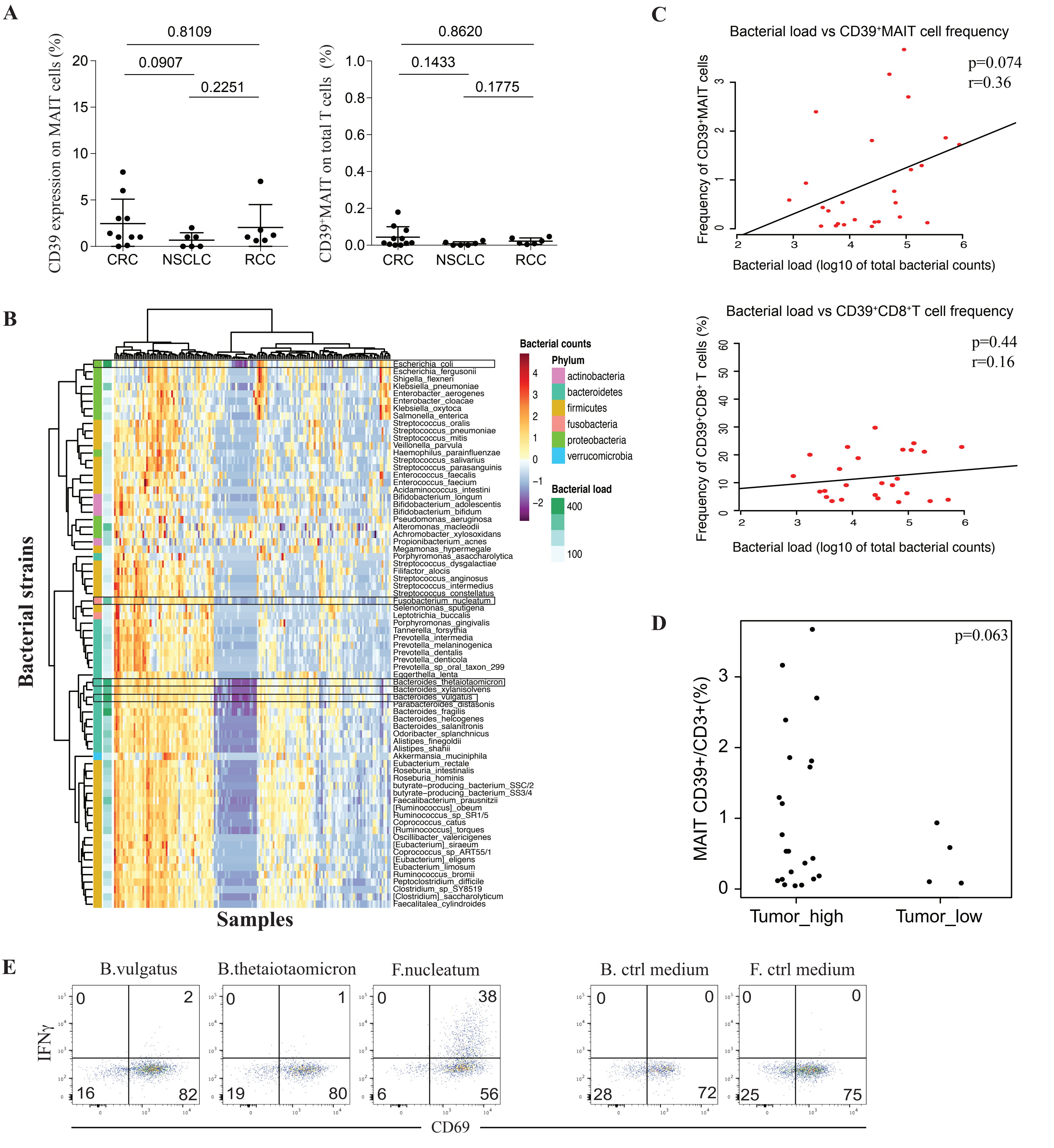

Supplementary figure S4: Extended figures of the microbiome-related analysis, related to Figure 4.

A. CD39 expression on MAIT cells (left) and on total T cells (right) in PBMC of CRC(n=10), NSCLC(n=6), RCC(n=6). Data are mean with S.D. from at least 7 experiments. Mann-Whitney U test. **B.** Bi-clustering of tumor samples (n=155) vs. selected bacterial species (n=74) after prefiltering. The number of species was cut down from 1479 to 74 based on the bacterial load across all tumor samples (>100). Each row represents one bacterial species, each column one tumor sample. The bacteria species were also classified according to the phylum they belong to (see legends). **C.** Correlation of relative bacterial load summed across all species for each tumor with CD39^{+}MAIT cell frequency on total CD3^{+}T cells (upper panel) or CD39^{+}CD8^{+}T cell frequency on total CD3^{+}T cells (lower panel), n=26. **D.** CD39^{+}MAIT cell frequency in tumors that are highly (n=22) or hardly bacteria-infiltrated (n=4). p=0.063, non-parametric t-test. **E.** *In vitro* screening of *Bacteroides* and *Fusobacterium* strains in their capability to activate MAIT cells. Culture media are used as controls.

Supplementary Figure S5

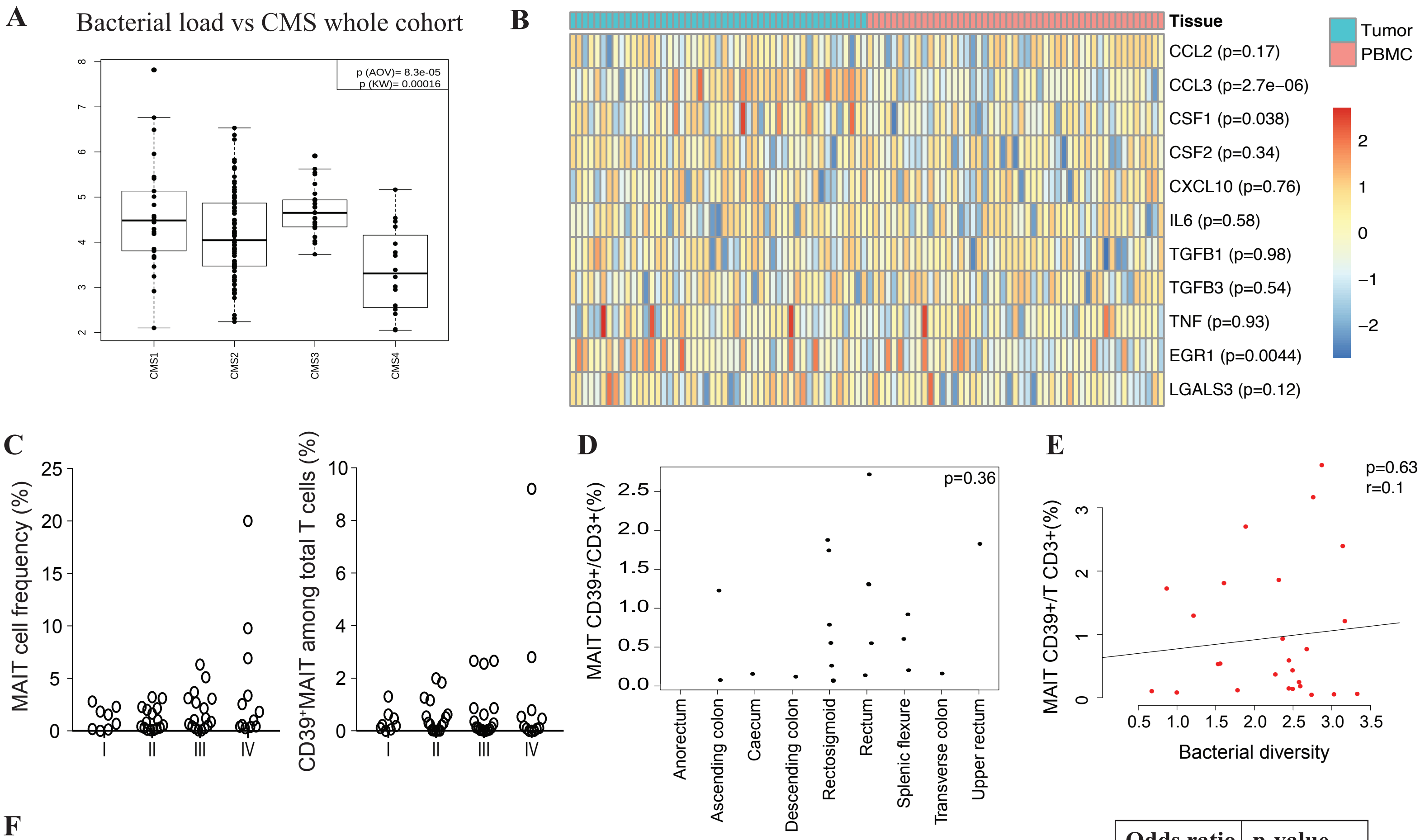

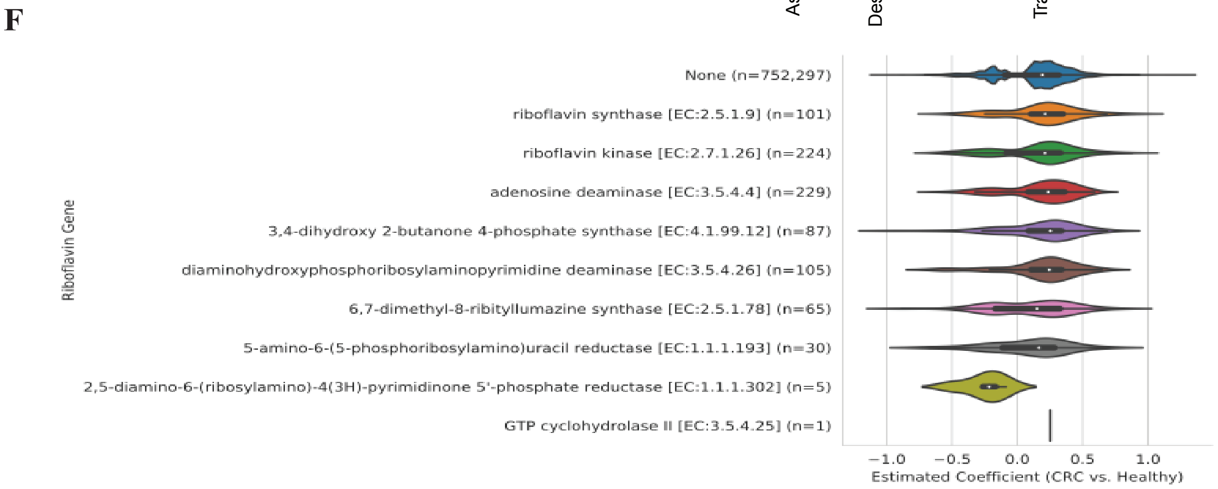

**Odds ratio**	**p-value**
-	-
1.47	0.12
1.08	0.66
1.35	0.07
1.21	0.48
**2**	**0.0063**
0.62	0.07
0.77	0.54
0.00	0.0017
-	-

Supplementary figure S5: Clinical associations in CRC, related to Figure 4.

A. Integration of the bacterial load obtained for each sample of the cohort to the consensus molecular classification of CRC previously described (36, 37). **B.** Heatmap of tissue-repair associated genes from scRNAseq profiling of MAIT cells in CRC tumor (blue) vs. healthy donor PBMC (red). Wilcoxon Mann-Whitney test. **C.** Frequency of total MAIT and $CD39^{+}$MAIT on total T cells in CRC tumor samples according to the disease stage (I, II, III or IV). n=8 for stage I, 16 for stage II, 17 for stage III and 12 for stage IV. **D**. Association between $CD39^{+}$MAIT cell frequency and tumor localization, n=21. **E.** Correlation between bacterial diversity and $CD39^{+}$MAIT cell frequency among total $CD3^{+}$ T cells, n=26. **F.** Violin plot showing distributions of the estimated coefficients in CRC vs. healthy metagenome for different microbiome-encoded genes involved in riboflavin metabolism. Analysis performed from data of *Minot et al, Microbiome, 2019* (40) (see methods).

Data S1

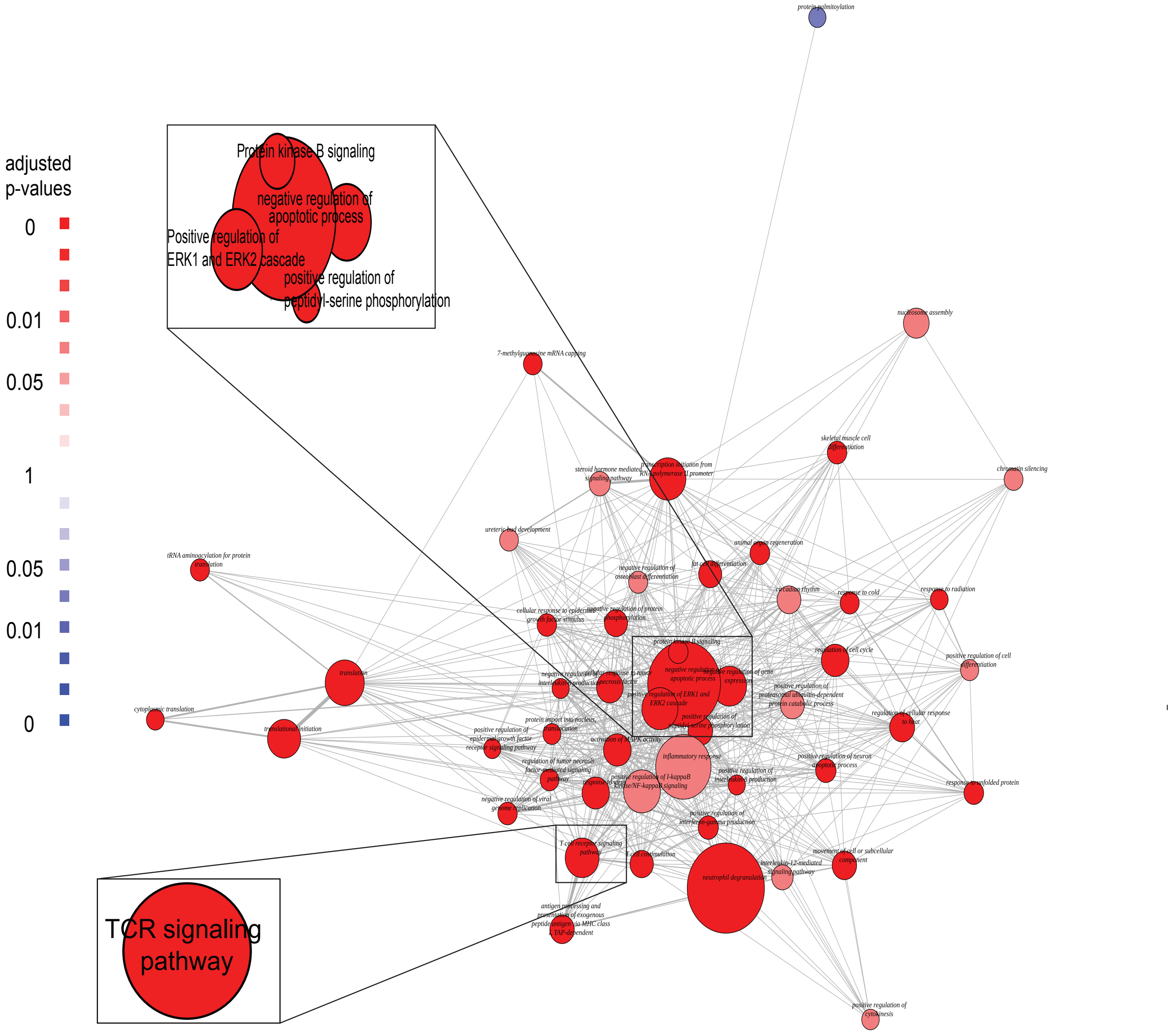

Data S1: Tumor-infiltrating MAIT cells express more apoptotic signals and a higher TCR signaling, related to Figure 1.

Gene enrichment map of MAIT cells in the tumors as compared to the paired PBMCs (n=2).
