# Supplementary material for: Human Tumor-Infiltrating MAIT Cells Display Hallmarks of Bacterial Antigen Recognition in Colorectal Cancer
Source: Cell Rep Med. 2020 Jun 23;1(3):100039. doi: 10.1016/j.xcrm.2020.100039 (PMC7659584; doi:10.1016/j.xcrm.2020.100039)
Supplement: Document S2. Article plus Supplemental Information [file mmc2.pdf]

Cell Reports Medicine

Report

Human Tumor-Infiltrating MAIT Cells Display Hallmarks of Bacterial Antigen Recognition in Colorectal Cancer

Graphical Abstract

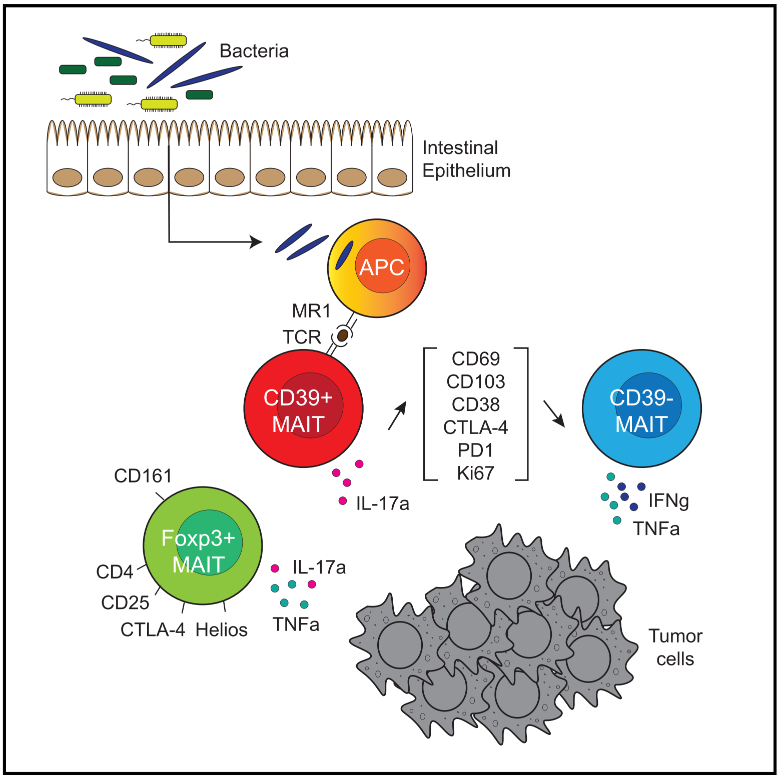

Highlights

- Comprehensive profiling of tumor-infiltrating MAIT cells in colorectal cancer (CRC)
- Identification of a tumor-infiltrating MAIT cell subset expressing CD4 and Foxp3
- TCR-driven CD39 expression is enriched in tumor-infiltrating MAIT cells
- CRC-related *Fusobacterium nucleatum* can activate MAIT cells in a TCR-dependent way

Authors

Shamin Li, Yannick Simoni, Etienne Becht, ..., Daniel S.W. Tan, Iain B. Tan, Evan W. Newell

Correspondence

shaminli@fredhutch.org (S.L.), enewell@fredhutch.org (E.W.N.)

In Brief

By investigating human colon, lung, and kidney carcinomas, Li et al. propose MAIT cells as part of the missing link between the gut microbiota and tumor biology. This work will open new avenues in cancer therapy by manipulating MAIT cells or MAIT-related bacterial ligands in the context of microbiota-influenced cancers.

Li et al., 2020, Cell Reports Medicine *1*, 100039
June 23, 2020 © 2020 The Authors.
https://doi.org/10.1016/j.xcrm.2020.100039

CellPress

Cell Reports Medicine

CellPress
OPEN ACCESS

Report

Human Tumor-Infiltrating MAIT Cells Display Hallmarks of Bacterial Antigen Recognition in Colorectal Cancer

Shamin Li,[1,*] Yannick Simoni,[1] Etienne Becht,[1] Chiew Yee Loh,[2] Naisi Li,[3] Daniel Lachance,[3] Si-Lin Koo,[4] Teck Por Lim,[5] Emile Kwong Wei Tan,[6] Ronnie Mathew,[6] Andrew Nguyen,[7] Justin Golovato,[7] Julia D. Berkson,[1] Martin Prlic,[1] Bernett Lee,[2] Samuel S. Minot,[8] Niranjan Nagarajan,[5,9] Neelendu Dey,[3] Daniel S.W. Tan,[4] Iain B. Tan,[4] and Evan W. Newell[1,10,*]

[1]Vaccine and Infectious Diseases Division, Fred Hutchinson Cancer Research Center, Seattle, WA, USA
[2]Agency for Science, Technology and Research (A*STAR), Singapore Immunology Network (SIgN), Singapore, Singapore
[3]Clinical Research Division, Fred Hutchinson Cancer Research Center, Seattle, WA, USA
[4]Division of Medical Oncology, National Cancer Centre Singapore (NCCS), Singapore, Singapore
[5]Computational and Systems Biology, Genome Institute of Singapore, Singapore, Singapore
[6]Department of Colorectal Surgery, Singapore General Hospital, Singapore, Singapore
[7]NantOmics, Santa Cruz, CA, USA
[8]Microbiome Research Initiative, Fred Hutchinson Cancer Research Center, Seattle, WA, USA
[9]Yong Loo Lin School of Medicine, National University of Singapore, Singapore, Singapore
[10]Lead Contact
*Correspondence: shaminli@fredhutch.org (S.L.), enewell@fredhutch.org (E.W.N.)
https://doi.org/10.1016/j.xcrm.2020.100039

SUMMARY

Growing evidence indicates a role for the gut microbiota in modulating anti-tumor treatment efficacy in human cancer. Here we study mucosa-associated invariant T (MAIT) cells to look for evidence of bacterial antigen recognition in human colon, lung, and kidney carcinomas. Using mass cytometry and single-cell mRNA sequencing, we identify a tumor-infiltrating MAIT cell subset expressing CD4 and Foxp3 and observe high expression of CD39 on MAIT cells from colorectal cancer (CRC) only, which we show *in vitro* to be expressed specifically after TCR stimulation. We further reveal that these cells are phenotypically and functionally exhausted. Sequencing data show high bacterial infiltration in CRC tumors and highlight an enriched species, *Fusobacteria nucleatum,* with capability to activate MAIT cells in a TCR-dependent way. Our results provide evidence of a MAIT cell response to microbial antigens in CRC and could pave the way for manipulating MAIT cells or the microbiome for cancer therapy.

INTRODUCTION

The era of cancer immunotherapy is in full swing, and different forms of treatment, such as checkpoint blockade immunotherapy, show uneven effects on restoring T cell immune responses. There is mounting evidence that the gut microbiota can strongly influence the antitumor efficacy of drugs (reviewed in Helmink et al.[1]). The first studies suggesting an immunotherapeutic effect of the microbiome showed improved effects of tumor-specific T cells by transferring bacterial products from the intestinal lumen to secondary lymphoid organs.[2] Ever since, the composition of the gut microbiota has been reported to influence anti-cancer therapeutic responses, including immune checkpoint blockade therapies targeting CTLA-4 and PD-1 (reviewed in Helmink et al.[1]). However, little is known about the mechanisms behind the varying responses.

Mucosa-associated invariant T (MAIT) cells are part of the unconventional or innate-like T cell family. These cells recognize bacterial antigens presented through MR1, a non-classical major histocompatibility complex (MHC) class I-like molecule. Mainly resident in the mucosa, MAIT cells are also found in lymphoid tissues and organs such as the liver and can account for up to 10% of total T cells in peripheral blood.[3–7] In humans, MAIT cells express the invariant T cell receptor (TCR) Vα7.2 associated with Jα33, Jα12, or Jα20. The TCR-β chains are more diverse, with a bias toward Vβ2 and Vβ13.[3,4,8,9] In 2012, identification of riboflavin precursor derivatives as the microbial ligands for MAIT cells allowed synthesis of MR1 tetramers to specifically identify these cells.[10,11] Most human MAIT cells are $CD8^+$, express the effector/memory phenotype $CD45RO^+$ $CD62L^{lo}$ $CCR7^-$, and are IL-$18R^{hi}$ [3]. They are known to protect against bacterial infection, are reported to be depleted during viral infection, and are implicated in several autoimmune diseases, including diabetes.[12–14]

In human cancers, the first report documenting the presence of MAIT cells showed that TRAV1-2 (coding for Vα7.2) and TRAJ33 (coding for Jα33) transcripts are enriched in brain and kidney tumors.[15] Other studies have shown an accumulation of

© 2020 The Authors. This is an open access article under the CC BY license (http://creativecommons.org/licenses/by/4.0/).

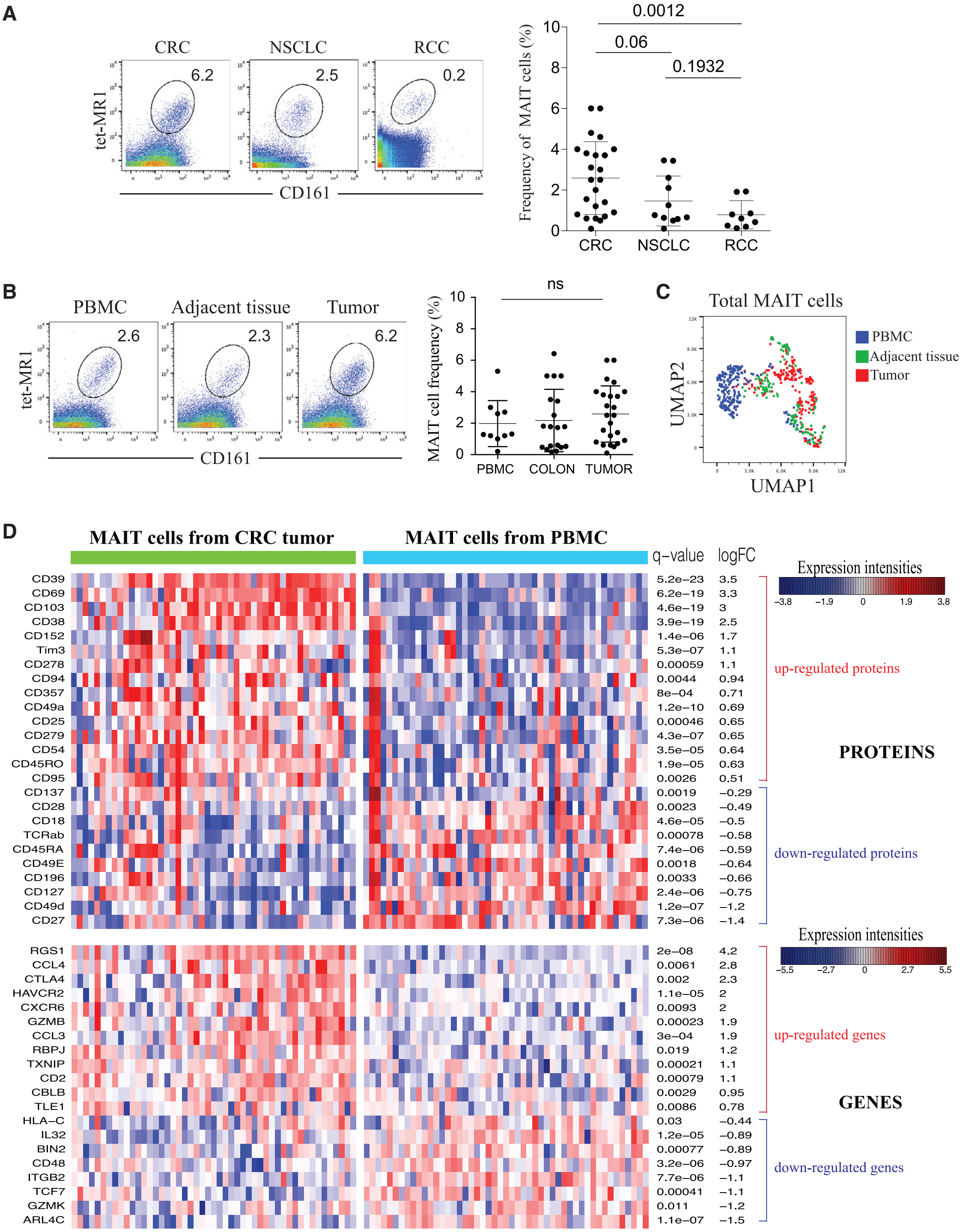

Figure 1. Tumor-Infiltrating MAIT Cells from CRC Show a Distinct Protein and Gene Profile

(A) Representative mass cytometry staining of MAIT cells in CRC, NSCLC, and RCC, gated on CD45^{+} live, DNA^{+}, CD14^{-}CD16^{-} CD3^{+} T cells (left) and frequencies of MAIT cells in the different tumors. CRC = 24, NSCLC = 11, RCC = 9 (right). Data are mean with SD from at least 10 experiments. Mann-Whitney U test.

(B) Representative MAIT cell staining from PBMC, adjacent tissue, and tumor of CRC, gated on total T cells. Shown are frequencies of MAIT cells in different compartments. PBMC = 10, colon = 19, tumor = 19. Data are mean with SD from at least 7 experiments. Mann-Whitney U test.

(C) UMAP plot of total MAIT cells from 2 PBMCs, 7 adjacent tissues, and 7 tumors of the same experiment.

(legend continued on next page)

these cells in colon adenocarcinoma with a reduced capability to produce interferon γ (IFNγ) and a positive correlation between a high tumor-infiltrating MAIT cell ratio and poor patient outcome (reviewed in Haeryfar et al.[16]). These results seem to indicate a negative effect of MAIT cell infiltration in tumors, but the function of these cells in the tumor microenvironment is lacking.

Because the riboflavin synthesis pathway is broadly conserved among many species of bacteria, MAIT cells can respond to a wide array of microbes, including known commensals.[10,17] Some groups have reported functional heterogeneity of MAIT cells as they respond to different bacterial or fungal organisms and adapt their antimicrobial response patterns.[18] In this regard, a recent study has assessed the ability of a large variety of commensal bacteria to activate MAIT cells in *in vitro* functional assays through human T cells engineered for MAIT TCRs.[18] These studies showed a potential effect of bacteria in shaping the function of MAIT cells under pathophysiological conditions. Here we hypothesize that MAIT cell responses can be initiated and modulated by gut microbiome-generated antigens in the tumor microenvironment. We aim to discern the role of MAIT cells at the interface between mucosa-associated cancers and the human gut microbiome by profiling colorectal cancer (CRC), non-small cell lung carcinoma (NSCLC), and renal cell carcinoma (RCC).

RESULTS

Tumor-Infiltrating MAIT Cells from CRC Show a Distinct Protein and Gene Profile

We first analyzed the frequency of MAIT cells in tumor samples from CRC, NSCLC, and RCC patients by mass cytometry (also known as CyTOF; STAR Methods). To ensure the robustness of our 5-OP-RU MR1 tetramer staining, we used Vα7.2 to confirm the specificity of 5-OP-RU MR1 and 6-FP MR1 to verify the absence of unspecific staining (Figures S1A and S1B). We observed that MAIT cells accounted for a higher proportion of total T cells in CRC compared with NSCLC and RCC (Figure 1A). No clear difference was detected in peripheral MAIT cell frequency between the three cancer types, indicating that the high infiltration of MAIT cells in CRC was tumor specific (Figure S1C). Using a 39-parameter panel, we focused our analysis on profiling tumor-infiltrating MAIT cells from CRC compared with PBMC and healthy adjacent tissue used as references. Although no difference was observed in MAIT cell frequency (Figure 1B), our analysis revealed a distinct phenotype of MAIT cells derived from tumor versus adjacent tissue or PBMC[19] (Figures 1C and S1D). At the gene level, bulk RNA sequencing of sorted MAIT cells showed a distinct transcriptomic profile between blood-circulating and tumor-infiltrating MAIT cells (Figure S1E). Specifically, gene set enrichment analysis (GSEA) highlighted an enrichment of TCR signaling and negative apoptotic regulation pathways from tumor-infiltrating MAIT cells (Figures S1F and S1G; Data S1). To further profile MAIT cells from CRC, we sorted MAIT cells from tumors and performed single-cell targeted mRNA sequencing (scRNAseq) in parallel with protein expression profiling using AbSeq on the BD Rhapsody system (STAR Methods).[20]) MAIT cells from healthy donor (HD) PBMC were analyzed simultaneously as a reference. We confirmed distinct protein and gene profiles in MAIT cells derived from tumors and PBMC (Figure 1D). Tumor-infiltrating MAIT cells highly expressed CD69, CD103, CD38, and CD39 with lower expression of CD27 and CD49d compared with peripheral MAIT cells. At the gene level, most tumor-infiltrating MAIT cells expressed CCL4, CCL3, and RGS1, indicating a high response to inflammation (Figure 1D). Moreover, these data revealed a heterogeneity among tumor-infiltrating MAIT cells from CRC that was not observed in peripheral MAIT cells. For instance, we detected the presence of CD39^{+} and CD39^{-} populations, each expressing a specific protein and transcriptomic signature. In the CD39^{+} population, we also distinguished subsets with unique protein and gene expression (CD69^{+}, CD103^{+}, and CD38^{+} versus CD152^{+}, Tim3^{+}, CD357^{+}, and CD45RA^{+}).

Identification of a CD4^{+} Foxp3^{+} Subset in Tumor-Infiltrating MAIT Cells

From the previous CyTOF analysis, the UMAP plot revealed a distinct cluster of CD4^{+} MAIT cells, some of which surprisingly co-expressed CTLA-4 and Foxp3 (Figure 2A). As shown with biaxial plots, we identified a population of CD4^{+} Foxp3^{+} tumor-infiltrating MAIT cells and validated them by flow cytometry on additional CRC tumor infiltrate samples (Figures 2B and S2A). No Foxp3^{+} MAIT cells were detected in the PBMCs of these patients, and only a few were observed in adjacent non-tumor tissue samples (Figure 2C). The frequency of CD4^{+} Foxp3^{+} MAIT cells in the tumors was heterogeneous and varied from 1% to 14% of total MAIT cells (Figure 2C). We found high expression of Helios, CTLA-4, and CD25 along with low expression of CD127 in these cells, similar to conventional Treg cells and in contrast to Foxp3^{-} MAIT cells (Figure 2D). These observations were confirmed by our scRNA-seq data, where few tumor-infiltrating MAIT cells expressing FOXP3 and co-expressing Treg-related surface markers were detected (Figure S2B). Although an association was observed between frequencies of CD4^{+} Foxp3^{+} MAIT cells and CD4^{+} MAIT cells, only a weak correlation was found between these cells and classical Tregs (Figures 2E and S2C). Functional analyses revealed an ability of CD4^{+} Foxp3^{+} MAIT cells to produce the pro-inflammatory cytokine tumor necrosis factor alpha (TNF-α), in contrast to Treg cells (Figure 2F). This result, supported by our scRNA-seq data, suggested that FoxP3 expression by tumor-infiltrating CD4^{+} MAIT cells was an indicator of activation rather than a marker of an immunosuppressive or regulatory subset (Figure S2D).[21]

(D) Heatmap showing differences in protein and gene expression between MAIT cells from CRC tumors and control healthy PBMC. Shown are only proteins and genes that are differentially expressed, with a fold-change of ±0.25 or more and adjusted p value (q value) of 0.05 or less. ScRNA-seq combined with the BD AbSeq Rhapsody system on sorted MAIT cells gated on live T cells using MR1 tetramer (STAR Methods).
See also Figure S1 and Data S1.

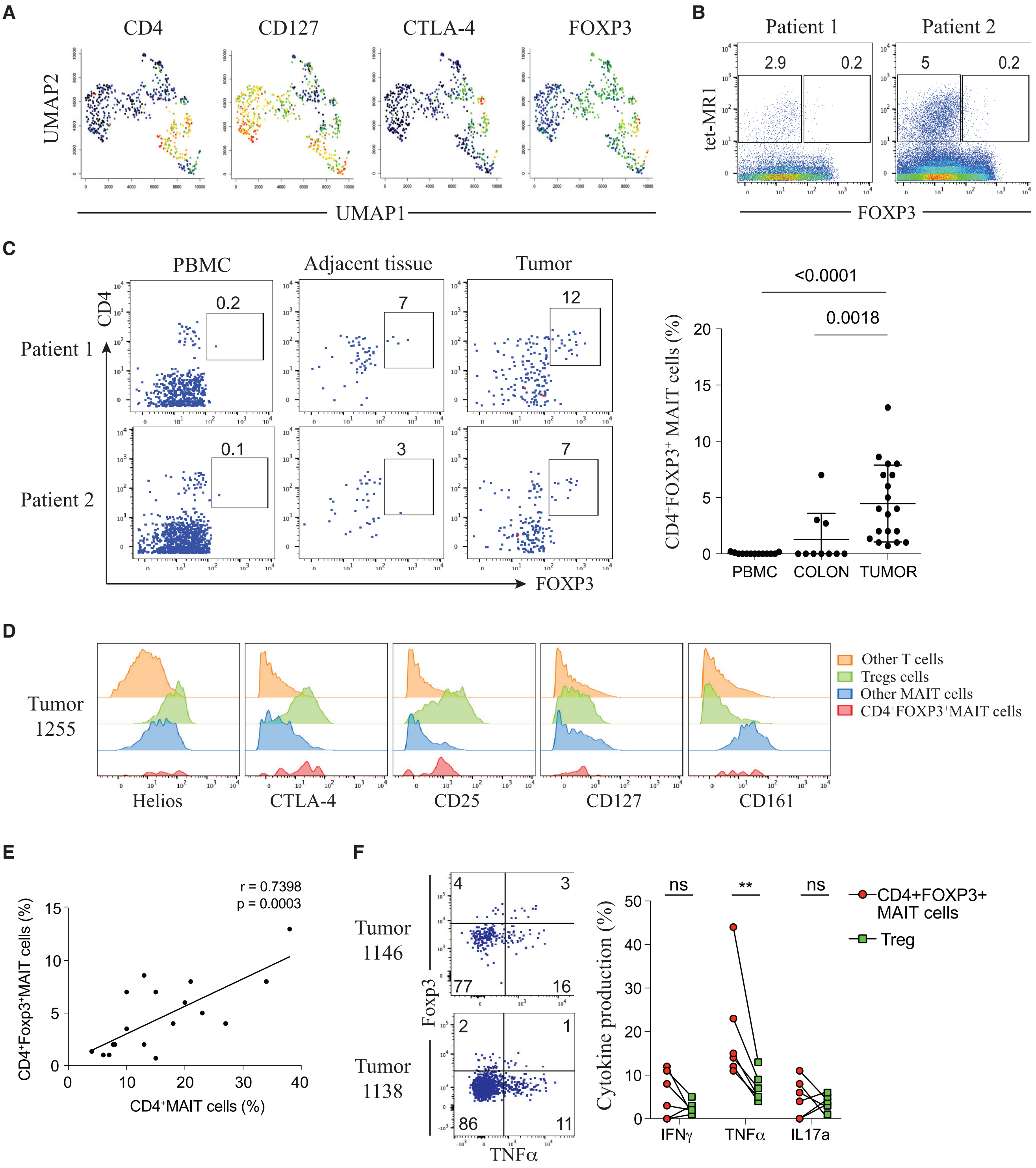

Figure 2. Identification of a CD4+ Foxp3+ Subset in Tumor-Infiltrating MAIT Cells

(A) UMAP plots showing expression of selected markers on MAIT cells; intensities are red (high), yellow/green (intermediate), blue (low).

(B) Tet-MR1 staining plotted against Foxp3 on total T cells from two CRC tumor samples.

(C) Representative staining of CD4+ Foxp3+ MAIT cells from PBMC, adjacent tissue, and tumors of two patients, gated on total MAIT cells. Shown are frequencies of Foxp3 expression among total MAIT cells (PBMC = 13, colon = 10, tumor = 19). Data are mean with SD from at least 7 experiments. Mann-Whitney U test.

(legend continued on next page)

Enrichment of CD39 Expression on Tumor-Infiltrating MAIT Cells

From our CyTOF analysis and supported by our scRNA-seq data, we also observed enrichment of CD39 expression on tumor-infiltrating MAIT cells (Figures 1D and 3A). CD39 was expressed at lower frequencies in the adjacent tissue and was almost absent in PBMCs (Figure 3A). In line with a previous report by our group,[22] we also observed a high frequency of $CD39^+$ $CD8^+$ tumor-infiltrating lymphocytes (TILs) in CRC (tet-MR1-negative T cells; Figure 3A). Although $CD39^+$ MAIT cells strongly expressed the tissue-resident markers CD103 and CD69, KLRG1 and interleukin-7R (IL-7R) expression was reduced on these cells compared with the $CD39^-$ population (Figures 3B and S3A). The inhibitory receptors PD-1 and CTLA-4 displayed elevated expression on the $CD39^+$ subset (Figures 3B and 3C), supporting the previous scRNA-seq data, which identified higher expression of genes associated with exhaustion on tumor-infiltrating $CD39^+$ MAIT cells, such as CTLA4 or HAVCR2 (Figure 1D). As we compared the phenotypic differences between MAIT cells from adjacent tissue and tumors, we also validated previous work reporting activation and tissue residency signatures for MAIT cells in human tissues (Figure S3B).[23,24] We then explored the functional characteristics of $CD39^+$ tumor-infiltrating MAIT cells. We observed that $CD39^+$ MAIT cells expressed less active caspase than the $CD39^-$ subset, suggesting protection against cell death (Figure S3C). Higher expression of Ki-67 was found in $CD39^+$ MAIT cells compared with their $CD39^-$ counterparts, indicating a higher proliferation rate (Figure 3D). We also evaluated the capability of these cells to produce cytokines, and our results indicated a lower polyfunctionality profile for $CD39^+$ MAIT cells (Figure 3E). In this regard, no impairment in expression of Granzyme A or B or perforin was observed for $CD39^+$ MAIT cells (Figure S3D). These cells showed higher expression of T-bet, PLZF, and Helios compared with the $CD39^-$ subset (Figure S3E). No clear association between non-MAIT $CD8^+$ $CD39^+$ TILs and $CD39^+$ MAIT cells was found, but a positive correlation was observed between the $CD4^+$ $Foxp3^+$ and $CD4^+$ $CD39^+$ MAIT subsets (Figure S3F). Previous studies reported CD39 to be expressed under chronic TCR stimulation on conventional T cells[25] and on tumor-specific $CD8^+$ T cells compared with cancer-unrelated $CD8^+$ T cells.[22,26,27] Because our results showed that tumor-infiltrating $CD39^+$ MAIT cells were associated with an exhausted profile and a lower apoptosis and higher proliferation rate, they could be undergoing antigen exposure and chronically activated through their TCR as well. Using *E. coli* described to specifically stimulate MAIT cells in a TCR-driven manner,[28] we performed *in vitro* assays and found that CD39 expression was induced after *E. coli* stimulation (Figure 3F). When stimulation was blocked with an anti-MR1 antibody, CD39 expression was strongly reduced, indicating TCR-dependent induction on peripheral MAIT cells. Interestingly, most $CD39^+$ MAIT cells were found to be negative for IFNγ expression, suggesting a dichotomy between TCR-dependent CD39 induction and cytokine production (Figure 3F). Although cytokine stimulation (IL-18 and IL-12) induced IFNγ production by MAIT cells, it failed to induce any expression of CD39, arguing against non-TCR-driven cytokine signaling (Figure 3F).

TCR-Dependent MAIT Cell Activation Is Associated with Tumor Infiltration by Bacteria

Based on these results, we hypothesized that tumor-infiltrating MAIT cell phenotype and function could be shaped by tumor-invasive and adherent bacteria via production of MAIT cell-agonistic metabolites. As described above, we observed differences in the extent to which MAIT cells were infiltrating CRC versus NSCLC or RCC. Based on the consensus that the microbial biomass from the colon is much higher than those from the lungs and kidneys,[29–32] we expected that colon tumors would be most infiltrated by bacteria, followed distantly by the lungs and then the kidneys. In light of this, we particularly found much higher CD39 expression on tumor-infiltrating MAIT cells from CRC (Figure 4A). In PBMC, we did not see significant changes in the frequency of $CD39^+$ MAIT cells across the three types of cancer (Figure S4A), supporting the idea that acquisition of CD39 expression was tumor specific. Of note, we observed a fairly conserved expression profile of tumor-infiltrating MAIT cells across the three tumor types, including high expression of tissue-resident and inhibitory markers, highlighting the specificity of CD39 expression on CRC tumor-infiltrating MAIT cells as being antigen specific (data not shown). To assess relationships between tumor infiltration by bacteria and the profiles of MAIT cells within CRC tumors, we analyzed data from whole-genome sequencing of tumor and paired adjacent tissue samples. Bacterial composition was determined using the metagenomic sequence classification software Kraken.[33] The bacterial load was higher in the tumor compared with the paired adjacent tissue (Figure 4B), which fits our observation of elevated CD39 expression by MAIT cells in tumors compared with healthy adjacent tissue (Figures 3A and S3B). Further analysis of the bacterial load in tumor versus adjacent tissue highlighted a few bacterial strains that were particularly enriched in these tumors, including *Escherichia coli* (Figure 4C). Because most bacterial species were barely detected, we focused our analysis on species with the highest abundance (more than 100 $\log_{10}$ counts across all tumor samples) and generated a biclustering graph of relative bacterial loads across tumor samples only. Using hierarchical clustering, we observed that the bacterial load across tumors was heterogeneous and that the abundances of bacteria from the same phylum were overall correlated (Figure S4B). From a subset of tumors for which MAIT cell phenotyping and whole-genome sequencing data were available (n = 26), we found that the frequency of $CD39^+$ MAIT but not $CD39^+CD8^+$ cells in tumors was fairly correlated

(D) Expression intensities of Treg-related markers and CD161 on different T cells compared with the $CD4^+$ $Foxp3^+$ MAIT subset; one representative tumor sample.
(E) Correlation of Foxp3 expression on MAIT cells with $CD4^+$ MAIT cell frequency; n = 20, two-tailed paired t test, Pearson's correlation.
(F) Co-expression of Foxp3 and TNF-α gated on total MAIT cells (left) and cytokine production (IFNγ, TNF-α, and IL-17) by $CD4^+$ $Foxp3^+$ tumor-infiltrating MAIT cells compared with Tregs upon 4 h of PMA/ionomycin stimulation (right); n = 6. Data are mean with SD from 2 experiments; two-tailed paired t test.
See also Figure S2.

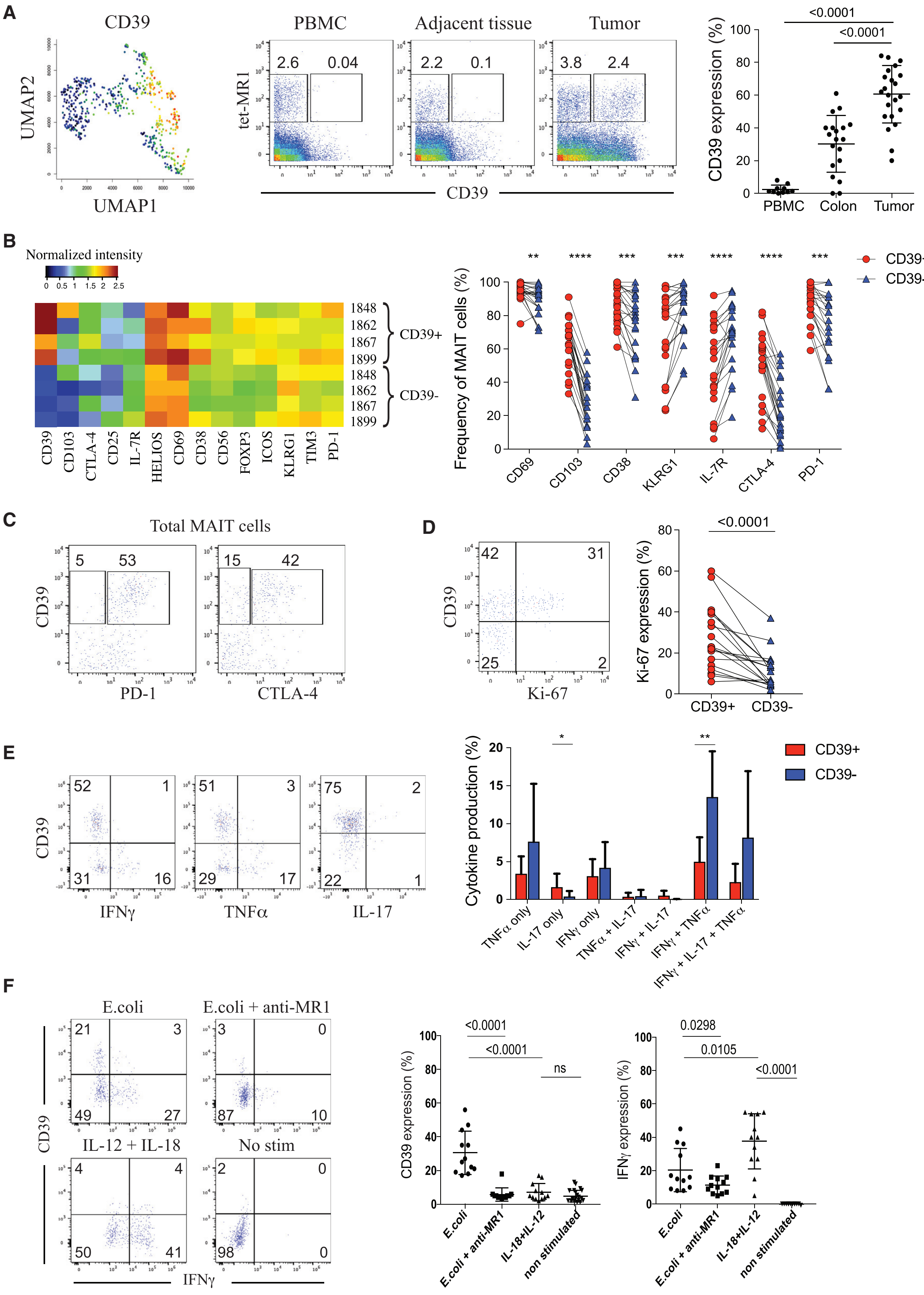

(legend on next page)

with the bacterial load obtained from the same patient (Figure S4C). As an additional analysis to support our hypothesis of CD39 expression on MAIT cells associated with tumor bacterial infiltration, we compared the percentage of CD39$^+$ MAIT cells in highly bacterially infiltrated tumors versus the ones in the middle of the biclustering graph displaying a low bacterial load (Figure S4B). Despite the low number of samples with data on CD39 expression, we observed that, overall, tumors with low bacterial infiltration showed lower levels of CD39 expression on MAIT cells (Figure S4D).

We next hypothesized that certain gut bacterial strains associated with CRC could modulate MAIT cell function. From the bacteria identified in Figures 4C and S4B, we selected few strains of *Bacteroides* and *Fusobacterium* that were present in tumors with a high load for *in vitro* stimulation assays. In particular, *Fusobacterium nucleatum* is widely reported to correlate with CRC pathogenesis.[34–38] We co-cultured *Bacteroides thetaiotaomicron*, *Bacteroides vulgatus*, or *Fusobacterium nucleatum* with HD PBMCs and observed that only *F. nucleatum* was able to activate MAIT cell and stimulate cytokine production (Figure S4E). Compared with different co-culture conditions, both live bacteria and culture supernatants from *F. nucleatum* were able to activate MAIT cells (Figure 4D). Anti-MR1 blocking assays dramatically reduced IFNγ production, arguing that MAIT cell activation by *F. nucleatum* was mediated through a TCR-dependent signal (Figure 4D). These results implicate *F. nucleatum* as a potential bacterial modulator of MAIT cell response in CRC.

Because it would be important to establish the role of tumor-infiltrating MAIT cells in CRC, we explored some of the defined MAIT cell features in the context of patient clinical outcome. First, based on the consensus molecular classification (CMS) used in CRC,[39,40] we found significant effects of CMS on bacterial composition, with the highest bacterial infiltration in the CMS3 subtype, suggesting a low immune and inflammatory signature (Figure S5A). We then considered expression of genes involved in tissue repair and wound healing functions for MAIT cells[41,42] and found that CCL3, CSF1, and EGR1 were significantly more expressed in tumor-infiltrating MAIT cells versus PBMC, arguing for a role of tumor-infiltrating MAIT cells in promoting epithelial growth (Figure S5B). Despite a trend toward a higher frequency of MAIT cells in later-stage CRC patients, our analysis related to different clinical parameters was not conclusive because of inadequate statistical power (Figures S5C–S5E). Finally, we explored the role of riboflavin biosynthesis gene expression in CRC prognosis and found that diaminohydroxyphosphoribosylaminopyrimidine deaminase (EC:3.5.4.26), also known as PyrD, was enriched in the CRC metagenome (Figure S5F; data from Minot and Willis[43]). Knowing that PyrD is necessary for MAIT cell development and activation,[44] this result might support the importance of MAIT cell infiltration and activation in CRC.

DISCUSSION

Our study aimed to decipher the role of MAIT cells in human cancer.[45–47] Although we observed a presence of MAIT cells in different cancer types, only MAIT cells from CRC were characterized by high expression of CD39, which we showed to be induced in a TCR-dependent manner. In line with our findings, a recent work associated chronic TCR stimulation and decreased responsiveness of gut MAIT cells with upregulation of PD-1 and CD39 expression.[48] We also identified a Foxp3$^+$ MAIT cell subset in tumors with a surface marker expression pattern similar to conventional Treg cells. The finding that they produced TNF-α and, to a lesser extent, IL-17 suggests pro-inflammatory potential. MAIT cells may possess an IL-17-producing subset derived from the periphery upon TCR activation in the presence of a pro-inflammatory milieu, as seen previously for non-MAIT CD4$^+$ FOXP3$^+$ T cells.[49] With this in mind, the higher IL-17 production observed by flow cytometry and expression of the inflammatory genes RSG1, CCL3, and CCL4 revealed by our scRNA-seq data could support a pro-tumorigenic role of CD39 expression in tumor-infiltrating MAIT cells in CRC.

We showed the capacity of *Fusobacterium nucleatum* to activate MAIT cells in a TCR-dependent manner. The reported high presence of this species in CRC versus NSCLC or RCC[50] strengthens the idea that TCR-mediated activation of MAIT cells depends on tumor microbiome composition and/or diversity. Although we believe in the potential role of *Fusobacterium nucleatum* in modulating the MAIT cell response in tumors, it is also conceivable that the latter could result from a synergistic response, given the substantial bacterial infiltration in CRC, and/or could be helped by additional mediators from the tumor microenvironment. Extended studies with tumor cultures or implementing colonic organoid systems could allow validation and further exploration of MAIT cells. This will help us to evaluate

Figure 3. High Enrichment of CD39 Expression on Tumor-Infiltrating MAIT Cells

(A) Left: CD39 expression shown by UMAP plot. Center: representative staining of CD39 expression on MAIT cells from PBMC, adjacent tissue, and tumor of CRC, gated on CD45$^+$ live, DNA$^+$, CD14$^-$ CD16$^-$ CD3$^+$ T cells. Right: frequencies of CD39$^+$ MAIT cells in different compartments. PBMC = 10, colon = 19, tumor = 24. Data are mean with SD from at least 7 experiments. Mann-Whitney U test.

(B). Expression intensities of selected markers on CD39$^+$ and CD39$^-$ MAIT cells in 4 different tumors (left). The numbers are patient IDs. Also shown is analysis of selected markers expression between the CD39$^+$ and CD39$^-$ subset; n = 22 (right). Data are from at least 7 experiments. Two-tailed paired t test.

(C) CD39 co-expression with PD-1 or CTLA-4 gated on total MAIT cells from one representative tumor sample.

(D) Representative staining of CD39 and Ki-67 expression on tumor-infiltrating MAIT cells (left) and Ki67 expression on CD39$^+$ and CD39$^-$ tumor-infiltrating MAIT cells (right); n = 18. Data are from at least 3 experiments. Two-tailed paired t test.

(E) Representative example of IFNγ, TNF-α, IL-17, and CD39 expression on tumor-infiltrating MAIT cells upon 4 h of PMA/ionomycin stimulation (left) and polyfunctionality profile of CD39$^+$ and CD39$^-$ tumor-infiltrating MAIT cells (right); n = 8. Data are mean with SD from 2 experiments. Two-tailed paired t test.

(F) Flow cytometry data of CD39 and IFNγ expression after different stimulation conditions, assessed on CD3$^+$ tet-MR1$^+$ CD161$^+$ cells; n = 12. Data are mean with SD from at least 3 experiments. Two-tailed paired t test.

See also Figure S3.

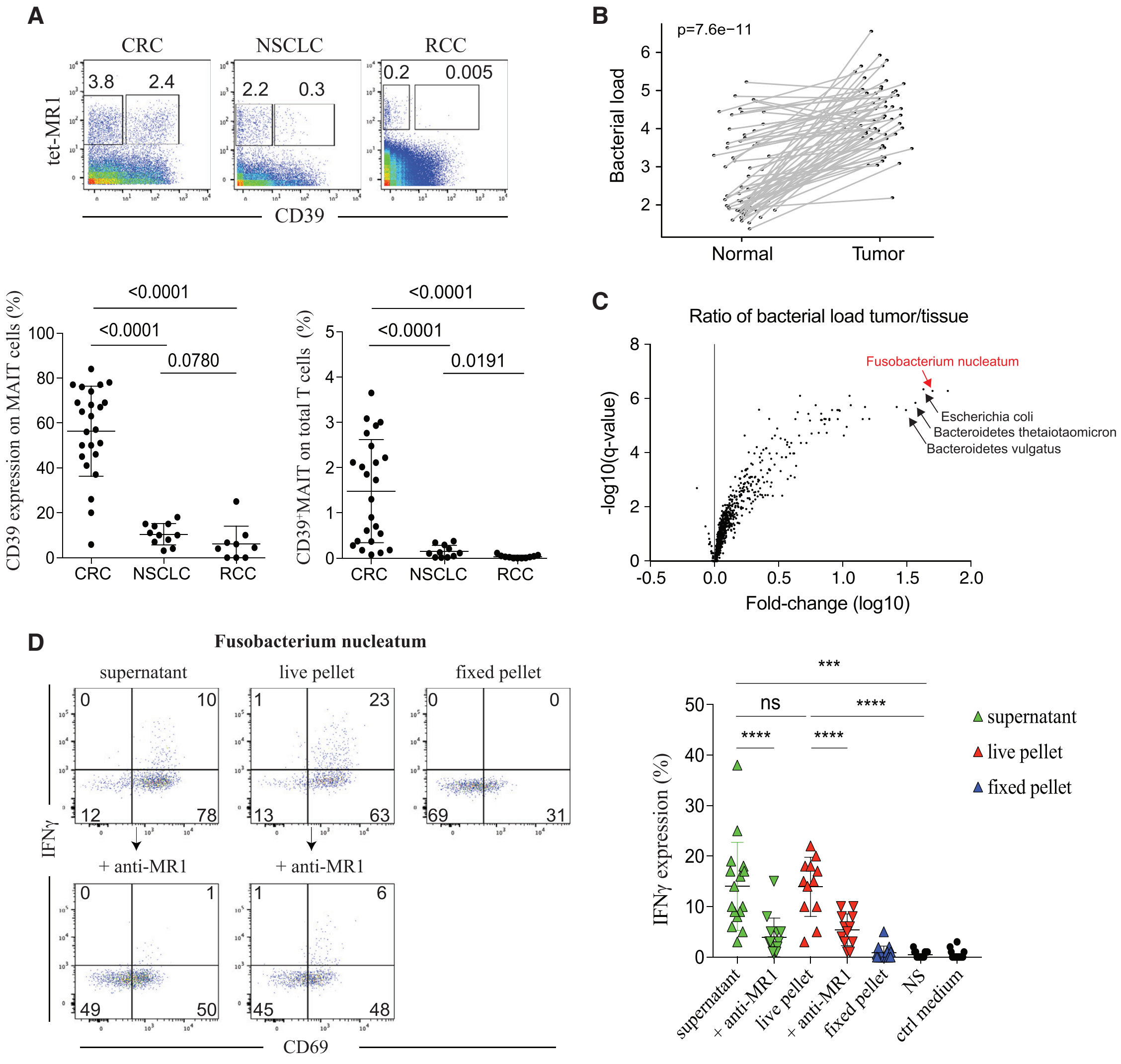

Figure 4. TCR-Dependent MAIT Cell Activation Is Associated with Tumor Infiltration by Bacteria

(A) Representative staining of CD39 expression plotted against tet-MR1 in CRC, NSCLC and RCC tumor samples, gated on total T cells (top panel). Frequencies of CD39 expression on total MAIT cells and CD39$^+$MAIT frequencies on total T cells (bottom panel). CRC = 24, NSCLC = 11, RCC = 9. Data are mean with SD from at least 10 experiments. Mann-Whitney U test.

(B) Relative bacterial load (per sample) summed across all species in adjacent tissue versus corresponding CRC tumors. n = 61, two-tailed paired t test.

(C) Ratio of of bacterial load in tumor versus tissue expressed in fold change(log10) versus p value expressed in -log10(q value). Bacterial species n = 1479.

(D) Flow cytometry data of IFNγ production by MAIT cells stimulated under different conditions (STAR Methods); n = 12–16. Data are mean with SD from 6 experiments. Two-tailed paired t test.

See also Figures S4 and S5.

the tumor specificity of this response and develop new tools for targeted cancer therapies.

Following a previous study of our group focusing on CD39$^+$ CD8$^+$ TILs,[22] we aimed to assess MAIT cells as antigen-specific versus innate-like bystanders in this context. Our results indicate that, from different aspects, CD39 expression on MAIT cells can be considered a marker of TCR-mediated antigen recognition and is associated with hallmarks of T cell exhaustion as in conventional CD8$^+$ T cells. So far, two reports have associated high infiltration of MAIT cells in tumors with a bad prognosis;[51,52] here we consider that several MAIT cell features possibly contribute to CRC development, including TCR-driven CD39 expression, tumor bacterial antigen recognition, pro-inflammatory signals, and riboflavin gene expression. Understanding what makes MAIT cells detrimental in the context of cancer is crucial, and unveiling their association with the tumor microbiome strongly supports the interest in manipulating the gut microbiome to reshape their response. Consequently, this study opens an avenue for modulating tumor immunity by targeting MAIT cells directly or indirectly through alterations in

the microbiome that would be designed to specifically modulate MAIT cell activity.

Limitations of Study

First, because of the limited number of Foxp3^{+} MAIT cells detected in the tumor infiltrate samples, additional studies will be needed to more properly assess the function of these cells. Second, because *in vitro* conditions do not fully mimic the tumor microenvironment and because MAIT cell ligands are highly unstable products,[11] it would be important to evaluate how bacterial dose- and/or timing-dependent stimulations can affect MAIT cell activation as well as differential expression of pertinent genes, differences in the concentration of the metabolite produced by the bacteria, etc. Last, and mostly because of inadequate statistical power, this study indicates but falls short of fully demonstrating the effect of CD39^{+}MAIT cells on the tumor microenvironment.

STAR★METHODS

Detailed methods are provided in the online version of this paper and include the following:

- KEY RESOURCES TABLE
- RESOURCE AVAILABILITY
  - Lead Contact
  - Materials Availability
  - Data and Code Availability
- EXPERIMENTAL MODEL AND SUBJECT DETAILS
  - Human samples
  - Bacterial strains
- METHOD DETAILS
  - Cell isolation
  - CyTOF staining
  - *in vitro* MAIT cell stimulation
  - FACS analysis
- QUANTIFICATION AND STATISTICAL ANALYSIS
  - CyTOF data analysis and UMAP
  - mRNA sequencing and Gene enrichment analysis
  - BD AbSeq single-cell mRNA sequencing
  - DNA extraction, WGS and metagenomic analysis
  - Expression of riboflavin genes in CRC metagenome

SUPPLEMENTAL INFORMATION

Supplemental Information can be found online at https://doi.org/10.1016/j.xcrm.2020.100039.

ACKNOWLEDGMENTS

The authors thank all members of former E.N. lab, SIgN, Singapore and the new lab at Fred Hutchinson Cancer Research Center, Seattle. We thank the postdocs in SIgN who provided help and insights. We also thank the members of the immunogenomic platform and the flow cytometry platform of SIgN and the clinical research coordinators from NCCS, Singapore. We thank Susan Bullman for helpful advice and insights. We thank the members of North-West BiosTrust (NWBT), who provided great help with collecting RCC samples, and the members of the cytometry and genomic platforms at Fred Hutch, Seattle. We greatly thank the NIH Tetramer Facility Core for providing the MR1 monomers. This study was funded by A-STAR/SIgN core funding (to E.W.N.), A-STAR/SIgN immunomonitoring platform funding (to E.W.N.), Fred Hutchinson Cancer Research Center new development funding (to E.W.N.), an Immunotherapy Integrated reseach center pilot grant (to E.W.N, M.P. and N.D.), partially funded by NIH grant R01AI123323 (to M.P.) and the National Medical Research Council (NMRC; Singapore) through the Translational and Clinical Research Program (NMRC/TCR/007-NCC/2013).

AUTHOR CONTRIBUTIONS

S.L. initiated and designed the project, performed the experiments, analyzed the data, and wrote the paper. Y.S. provided and helped process the samples, discussed the data, and reviewed the paper. E.B. helped to analyze the transcriptomics and whole-genome sequencing data, discussed the data, and reviewed the paper. C.Y.L. helped to process samples. N.L. and D.L. performed anaerobic gut bacterial culturing for the *in vitro* MAIT cell stimulation assays. S.-L.K., E.K.W.T., and R.M. recruited patients, facilitated sample collection, and accumulated clinical information. J.G. handled WGS sequencing from tissue to raw data. A.N. helped align and assemble WGS data. J.D.B. and M.P. provided scientific insights. B.L. helped with the transcriptomics data. T.P.L. and N.N. helped with the whole-genome sequencing data. S.S.M. provided scientific insights and performed the association analysis between riboflavin genes and the CRC metagenome with data published in Minot et al.[43] N.D. provided scientific insights and oversight of anaerobic gut bacterial culturing and reviewed the paper. D.S.W.T. provided the samples. I.B.T. recruited patients and reviewed the paper. E.W.N. led the project, developed scripts for CyTOF analysis, discussed the data, and wrote the paper.

DECLARATION OF INTERESTS

E.W.N is a co-founder, advisor, and shareholder of ImmunoScape Pte. Ltd. and an advisor for Neogene Therapeutics and Nanostring Technologies.

Received: November 26, 2019
Revised: March 25, 2020
Accepted: May 29, 2020
Published: June 23, 2020

REFERENCES

1. Helmink, B.A., Khan, M.A.W., Hermann, A., Gopalakrishnan, V., and Wargo, J.A. (2019). The microbiome, cancer, and cancer therapy. Nat. Med. *25*, 377–388.
2. Paulos, C.M., Wrzesinski, C., Kaiser, A., Hinrichs, C.S., Chieppa, M., Cassard, L., Palmer, D.C., Boni, A., Muranski, P., Yu, Z., et al. (2007). Microbial translocation augments the function of adoptively transferred self/tumor-specific CD8+ T cells via TLR4 signaling. J. Clin. Invest. *117*, 2197–2204.
3. Reantragoon, R., Corbett, A.J., Sakala, I.G., Gherardin, N.A., Furness, J.B., Chen, Z., Eckle, S.B., Uldrich, A.P., Birkinshaw, R.W., Patel, O., et al. (2013). Antigen-loaded MR1 tetramers define T cell receptor heterogeneity in mucosal-associated invariant T cells. J. Exp. Med. *210*, 2305–2320.
4. Lepore, M., Kalinichenko, A., Colone, A., Paleja, B., Singhal, A., Tschumi, A., Lee, B., Poidinger, M., Zolezzi, F., Quagliata, L., et al. (2014). Parallel T-cell cloning and deep sequencing of human MAIT cells reveal stable oligoclonal TCRβ repertoire. Nat. Commun. *5*, 3866.
5. Dusseaux, M., Martin, E., Serriari, N., Péguillet, I., Premel, V., Louis, D., Milder, M., Le Bourhis, L., Soudais, C., Treiner, E., and Lantz, O. (2011). Human MAIT cells are xenobiotic-resistant, tissue-targeted, CD161hi IL-17-secreting T cells. Blood *117*, 1250–1259.
6. Gibbs, A., Leeansyah, E., Introini, A., Paquin-Proulx, D., Hasselrot, K., Andersson, E., Broliden, K., Sandberg, J.K., and Tjernlund, A. (2017). MAIT cells reside in the female genital mucosa and are biased towards IL-17 and IL-22 production in response to bacterial stimulation. Mucosal Immunol. *10*, 35–45.

7. Tang, X.Z., Jo, J., Tan, A.T., Sandalova, E., Chia, A., Tan, K.C., Lee, K.H., Gehring, A.J., De Libero, G., and Bertoletti, A. (2013). IL-7 licenses activation of human liver intrasinusoidal mucosal-associated invariant T cells. J. Immunol. *190*, 3142–3152.

8. Tilloy, F., Treiner, E., Park, S.H., Garcia, C., Lemonnier, F., de la Salle, H., Bendelac, A., Bonneville, M., and Lantz, O. (1999). An invariant T cell receptor alpha chain defines a novel TAP-independent major histocompatibility complex class Ib-restricted alpha/beta T cell subpopulation in mammals. J. Exp. Med. *189*, 1907–1921.

9. Porcelli, S., Yockey, C.E., Brenner, M.B., and Balk, S.P. (1993). Analysis of T cell antigen receptor (TCR) expression by human peripheral blood CD4-8- alpha/beta T cells demonstrates preferential use of several V beta genes and an invariant TCR alpha chain. J. Exp. Med. *178*, 1–16.

10. Kjer-Nielsen, L., Patel, O., Corbett, A.J., Le Nours, J., Meehan, B., Liu, L., Bhati, M., Chen, Z., Kostenko, L., Reantragoon, R., et al. (2012). MR1 presents microbial vitamin B metabolites to MAIT cells. Nature *491*, 717–723.

11. Corbett, A.J., Eckle, S.B., Birkinshaw, R.W., Liu, L., Patel, O., Mahony, J., Chen, Z., Reantragoon, R., Meehan, B., Cao, H., et al. (2014). T-cell activation by transitory neo-antigens derived from distinct microbial pathways. Nature *509*, 361–365.

12. Howson, L.J., Salio, M., and Cerundolo, V. (2015). MR1-Restricted Mucosal-Associated Invariant T Cells and Their Activation during Infectious Diseases. Front. Immunol. *6*, 303.

13. van Wilgenburg, B., Scherwitzl, I., Hutchinson, E.C., Leng, T., Kurioka, A., Kulicke, C., de Lara, C., Cole, S., Vasanawathana, S., Limpitikul, W., et al.; STOP-HCV consortium (2016). MAIT cells are activated during human viral infections. Nat. Commun. *7*, 11653.

14. Rouxel, O., Da Silva, J., Beaudoin, L., Nel, I., Tard, C., Cagninacci, L., Kiaf, B., Oshima, M., Diedisheim, M., Salou, M., et al. (2017). Cytotoxic and regulatory roles of mucosal-associated invariant T cells in type 1 diabetes. Nat. Immunol. *18*, 1321–1331.

15. Peterfalvi, A., Gomori, E., Magyarlaki, T., Pal, J., Banati, M., Javorhazy, A., Szekeres-Bartho, J., Szereday, L., and Illes, Z. (2008). Invariant Valpha7.2-Jalpha33 TCR is expressed in human kidney and brain tumors indicating infiltration by mucosal-associated invariant T (MAIT) cells. Int. Immunol. *20*, 1517–1525.

16. Haeryfar, S.M.M., Shaler, C.R., and Rudak, P.T. (2018). Mucosa-associated invariant T cells in malignancies: a faithful friend or formidable foe? Cancer Immunol. Immunother. *67*, 1885–1896.

17. Le Bourhis, L., Martin, E., Péguillet, I., Guihot, A., Froux, N., Coré, M., Lévy, E., Dusseaux, M., Meyssonnier, V., Premel, V., et al. (2010). Antimicrobial activity of mucosal-associated invariant T cells. Nat. Immunol. *11*, 701–708.

18. Tastan, C., Karhan, E., Zhou, W., Fleming, E., Voigt, A.Y., Yao, X., Wang, L., Horne, M., Placek, L., Kozhaya, L., et al. (2018). Tuning of human MAIT cell activation by commensal bacteria species and MR1-dependent T-cell presentation. Mucosal Immunol. *11*, 1591–1605.

19. Becht, E., McInnes, L., Healy, J., Dutertre, C.A., Kwok, I.W.H., Ng, L.G., Ginhoux, F., and Newell, E.W. (2018). Dimensionality reduction for visualizing single-cell data using UMAP. Nat. Biotechnol. *37*, 38–44.

20. Mair, F.E.J.R., Voillet, V., Simoni, Y., Bi, T., Tyznik, A.J., Martin, J., Gottardo, R., Newell, E.W., and Prlic, M. (2019). A targeted multi-omic analysis approach measures protein expression and low abundance transcripts on the single cell level. Cell Rep. *31*, 107499.

21. Wang, J., Ioan-Facsinay, A., van der Voort, E.I., Huizinga, T.W., and Toes, R.E. (2007). Transient expression of FOXP3 in human activated nonregulatory CD4+ T cells. Eur. J. Immunol. *37*, 129–138.

22. Simoni, Y., Becht, E., Fehlings, M., Loh, C.Y., Koo, S.L., Teng, K.W.W., Yeong, J.P.S., Nahar, R., Zhang, T., Kared, H., et al. (2018). Bystander $CD8^+$ T cells are abundant and phenotypically distinct in human tumour infiltrates. Nature *557*, 575–579.

23. Schmaler, M., Colone, A., Spagnuolo, J., Zimmermann, M., Lepore, M., Kalinichenko, A., Bhatia, S., Cottier, F., Rutishauser, T., Pavelka, N., et al. (2018). Modulation of bacterial metabolism by the microenvironment controls MAIT cell stimulation. Mucosal Immunol. *11*, 1060–1070.

24. Salou, M., Legoux, F., Gilet, J., Darbois, A., du Halgouet, A., Alonso, R., Richer, W., Goubet, A.G., Daviaud, C., Menger, L., et al. (2019). A common transcriptomic program acquired in the thymus defines tissue residency of MAIT and NKT subsets. J. Exp. Med. *216*, 133–151.

25. Gupta, P.K., Godec, J., Wolski, D., Adland, E., Yates, K., Pauken, K.E., Cosgrove, C., Ledderose, C., Junger, W.G., Robson, S.C., et al. (2015). CD39 Expression Identifies Terminally Exhausted CD8+ T Cells. PLoS Pathog. *11*, e1005177.

26. Li, H., van der Leun, A.M., Yofe, I., Lubling, Y., Gelbard-Solodkin, D., van Akkooi, A.C.J., van den Braber, M., Rozeman, E.A., Haanen, J.B.A.G., Blank, C.U., et al. (2019). Dysfunctional CD8 T Cells Form a Proliferative, Dynamically Regulated Compartment within Human Melanoma. Cell *176*, 775–789.e18.

27. Duhen, T., Duhen, R., Montler, R., Moses, J., Moudgil, T., de Miranda, N.F., Goodall, C.P., Blair, T.C., Fox, B.A., McDermott, J.E., et al. (2018). Co-expression of CD39 and CD103 identifies tumor-reactive CD8 T cells in human solid tumors. Nat. Commun. *9*, 2724.

28. Dias, J., Leeansyah, E., and Sandberg, J.K. (2017). Multiple layers of heterogeneity and subset diversity in human MAIT cell responses to distinct microorganisms and to innate cytokines. Proc. Natl. Acad. Sci. USA *114*, E5434–E5443.

29. Lloyd-Price, J., Abu-Ali, G., and Huttenhower, C. (2016). The healthy human microbiome. Genome Med. *8*, 51.

30. Yu, G., Gail, M.H., Consonni, D., Carugno, M., Humphrys, M., Pesatori, A.C., Caporaso, N.E., Goedert, J.J., Ravel, J., and Landi, M.T. (2016). Characterizing human lung tissue microbiota and its relationship to epidemiological and clinical features. Genome Biol. *17*, 163.

31. O'Dwyer, D.N., Dickson, R.P., and Moore, B.B. (2016). The Lung Microbiome, Immunity, and the Pathogenesis of Chronic Lung Disease. J. Immunol. *196*, 4839–4847.

32. Pevsner-Fischer, M., Tuganbaev, T., Meijer, M., Zhang, S.H., Zeng, Z.R., Chen, M.H., and Elinav, E. (2016). Role of the microbiome in non-gastrointestinal cancers. World J. Clin. Oncol. *7*, 200–213.

33. Wood, D.E., and Salzberg, S.L. (2014). Kraken: ultrafast metagenomic sequence classification using exact alignments. Genome Biol. *15*, R46.

34. Castellarin, M., Warren, R.L., Freeman, J.D., Dreolini, L., Krzywinski, M., Strauss, J., Barnes, R., Watson, P., Allen-Vercoe, E., Moore, R.A., and Holt, R.A. (2012). Fusobacterium nucleatum infection is prevalent in human colorectal carcinoma. Genome Res. *22*, 299–306.

35. Kostic, A.D., Gevers, D., Pedamallu, C.S., Michaud, M., Duke, F., Earl, A.M., Ojesina, A.I., Jung, J., Bass, A.J., Tabernero, J., et al. (2012). Genomic analysis identifies association of Fusobacterium with colorectal carcinoma. Genome Res. *22*, 292–298.

36. Flanagan, L., Schmid, J., Ebert, M., Soucek, P., Kunicka, T., Liska, V., Bruha, J., Neary, P., Dezeeuw, N., Tommasino, M., et al. (2014). Fusobacterium nucleatum associates with stages of colorectal neoplasia development, colorectal cancer and disease outcome. Eur. J. Clin. Microbiol. Infect. Dis. *33*, 1381–1390.

37. Mima, K., Sukawa, Y., Nishihara, R., Qian, Z.R., Yamauchi, M., Inamura, K., Kim, S.A., Masuda, A., Nowak, J.A., Nosho, K., et al. (2015). Fusobacterium nucleatum and T Cells in Colorectal Carcinoma. JAMA Oncol. *1*, 653–661.

38. Bullman, S., Pedamallu, C.S., Sicinska, E., Clancy, T.E., Zhang, X., Cai, D., Neuberg, D., Huang, K., Guevara, F., Nelson, T., et al. (2017). Analysis of *Fusobacterium* persistence and antibiotic response in colorectal cancer. Science *358*, 1443–1448.

39. Guinney, J., Dienstmann, R., Wang, X., de Reyniès, A., Schlicker, A., Soneson, C., Marisa, L., Roepman, P., Nyamundanda, G., Angelino, P., et al. (2015). The consensus molecular subtypes of colorectal cancer. Nat. Med. *21*, 1350–1356.

CellPress
OPEN ACCESS

40. Becht, E., de Reyniès, A., Giraldo, N.A., Pilati, C., Buttard, B., Lacroix, L., Selves, J., Sautès-Fridman, C., Laurent-Puig, P., and Fridman, W.H. (2016). Immune and Stromal Classification of Colorectal Cancer Is Associated with Molecular Subtypes and Relevant for Precision Immunotherapy. Clin. Cancer Res. *22*, 4057–4066.

41. Linehan, J.L., Harrison, O.J., Han, S.J., Byrd, A.L., Vujkovic-Cvijin, I., Villarino, A.V., Sen, S.K., Shaik, J., Smelkinson, M., Tamoutounour, S., et al. (2018). Non-classical Immunity Controls Microbiota Impact on Skin Immunity and Tissue Repair. Cell *172*, 784, 96.e18.

42. Constantinides, M.G., Link, V.M., Tamoutounour, S., Wong, A.C., Perez-Chaparro, P.J., Han, S.J., Chen, Y.E., Li, K., Farhat, S., Weckel, A., et al. (2019). MAIT cells are imprinted by the microbiota in early life and promote tissue repair. Science *366*, eaax6624.

43. Minot, S.S., and Willis, A.D. (2019). Clustering co-abundant genes identifies components of the gut microbiome that are reproducibly associated with colorectal cancer and inflammatory bowel disease. Microbiome *7*, 110.

44. Kjer-Nielsen, L., Corbett, A.J., Chen, Z., Liu, L., Mak, J.Y., Godfrey, D.I., Rossjohn, J., Fairlie, D.P., McCluskey, J., and Eckle, S.B. (2018). An overview on the identification of MAIT cell antigens. Immunol. Cell Biol. *96*, 573–587.

45. Toubal, A., Nel, I., Lotersztajn, S., and Lehuen, A. (2019). Mucosal-associated invariant T cells and disease. Nat. Rev. Immunol. *19*, 643–657.

46. Godfrey, D.I., Koay, H.F., McCluskey, J., and Gherardin, N.A. (2019). The biology and functional importance of MAIT cells. Nat. Immunol. *20*, 1110–1128.

47. Provine, N.M., and Klenerman, P. (2020). MAIT Cells in Health and Disease. Annu. Rev. Immunol. *38*, 203–228.

48. Leng, T., Akther, H.D., Hackstein, C.P., Powell, K., King, T., Friedrich, M., Christoforidou, Z., Mccuaig, S., Neyazi, M., Arancibia-Carcamo, C.V., et al. (2018). TCR and inflammatory signals tune human MAIT cells to exert specific tissue repair and effector functions. Cell Rep. *28*, 3077–3091.e5.

49. Beriou, G., Costantino, C.M., Ashley, C.W., Yang, L., Kuchroo, V.K., Baecher-Allan, C., and Hafler, D.A. (2009). IL-17-producing human peripheral regulatory T cells retain suppressive function. Blood *113*, 4240–4249.

50. Routy, B., Le Chatelier, E., Derosa, L., Duong, C.P.M., Alou, M.T., Daillère, R., Fluckiger, A., Messaoudene, M., Rauber, C., Roberti, M.P., et al. (2018). Gut microbiome influences efficacy of PD-1-based immunotherapy against epithelial tumors. Science *359*, 91–97.

51. Zabijak, L., Attencourt, C., Guignant, C., Chatelain, D., Marcelo, P., Marolleau, J.P., and Treiner, E. (2015). Increased tumor infiltration by mucosal-associated invariant T cells correlates with poor survival in colorectal cancer patients. Cancer Immunol. Immunother. *64*, 1601–1608.

52. Duan, M., Goswami, S., Shi, J.Y., Wu, L.J., Wang, X.Y., Ma, J.Q., Zhang, Z., Shi, Y., Ma, L.J., Zhang, S., et al. (2019). Activated and Exhausted MAIT Cells Foster Disease Progression and Indicate Poor Outcome in Hepatocellular Carcinoma. Clin. Cancer Res. *25*, 3304–3316.

53. Dey, N., Wagner, V.E., Blanton, L.V., Cheng, J., Fontana, L., Haque, R., Ahmed, T., and Gordon, J.I. (2015). Regulators of gut motility revealed by a gnotobiotic model of diet-microbiome interactions related to travel. Cell *163*, 95–107.

54. Ganguli, L.A., Turton, L.J., and Tillotson, G.S. (1982). Evaluation of Fastidious Anaerobe Broth as a blood culture medium. J. Clin. Pathol. *35*, 458–461.

55. Newell, E.W., Sigal, N., Nair, N., Kidd, B.A., Greenberg, H.B., and Davis, M.M. (2013). Combinatorial tetramer staining and mass cytometry analysis facilitate T-cell epitope mapping and characterization. Nat. Biotechnol. *31*, 623–629.

56. Becher, B., Schlitzer, A., Chen, J., Mair, F., Sumatoh, H.R., Teng, K.W., Low, D., Ruedl, C., Riccardi-Castagnoli, P., Poidinger, M., et al. (2014). High-dimensional analysis of the murine myeloid cell system. Nat. Immunol. *15*, 1181–1189.

57. Finck, R., Simonds, E.F., Jager, A., Krishnaswamy, S., Sachs, K., Fantl, W., Pe'er, D., Nolan, G.P., and Bendall, S.C. (2013). Normalization of mass cytometry data with bead standards. Cytometry A *83*, 483–494.

58. Moore, W.A., and Parks, D.R. (2012). Update for the logicle data scale including operational code implementations. Cytometry A *81*, 273–277.

59. Harrow, J., Denoeud, F., Frankish, A., Reymond, A., Chen, C.K., Chrast, J., Lagarde, J., Gilbert, J.G.R., Storey, R., Swarbreck, D., et al. (2006). GENCODE: producing a reference annotation for ENCODE. Genome Biol. *7*, S4.1–S4.9.

60. Patro, R., Duggal, G., Love, M.I., Irizarry, R.A., and Kingsford, C. (2017). Salmon provides fast and bias-aware quantification of transcript expression. Nat. Methods *14*, 417–419.

61. Robinson, M.D., McCarthy, D.J., and Smyth, G.K. (2010). edgeR: a Bioconductor package for differential expression analysis of digital gene expression data. Bioinformatics *26*, 139–140.

62. Butler, A., Hoffman, P., Smibert, P., Papalexi, E., and Satija, R. (2018). Integrating single-cell transcriptomic data across different conditions, technologies, and species. Nat. Biotechnol. *36*, 411–420.

63. North, J.P., Golovato, J., Vaske, C.J., Sanborn, J.Z., Nguyen, A., Wu, W., Goode, B., Stevers, M., McMullen, K., Perez White, B.E., et al. (2018). Cell of origin and mutation pattern define three clinically distinct classes of sebaceous carcinoma. Nat. Commun. *9*, 1894.

64. Li, H., Ruan, J., and Durbin, R. (2008). Mapping short DNA sequencing reads and calling variants using mapping quality scores. Genome Res. *18*, 1851–1858.

STAR★METHODS

KEY RESOURCES TABLE

REAGENT or RESOURCE	SOURCE	IDENTIFIER
Antibodies		
89 - CD45 (clone HI30)	Fluidigm	Cat# 3089003, RRID:AB_2661851
112/114 - CD14 (clone TuK4)	Thermofisher	Cat# MHCD1400, RRID:AB_10371749
115 - CD57 (clone HCD57)	Biolegend	N/A
140 - CD28 (clone CD28.2)	Biolegend	Cat# 302902, RRID:AB_314304
140 - CD4 (clone RPA-T4)	Biolegend	Cat# 300502, RRID:AB_314070
141 - CD56 (clone NCAM16.2)	BD	Cat# 559043, RRID:AB_397180
142 - HLA-DR (clone L243)	Biolegend	Cat# 307602, RRID:AB_314680
143 - CD3 (clone UCHT1)	Biolegend	Cat# 300402, RRID:AB_314056
144- IL18R (clone H44)	Biolegend	Cat# 313804, RRID:AB_345312
145- CD69 (clone FN50)	Biolegend	Cat# 310902, RRID:AB_314837
146 - CD8 (clone RPA-T8)	Biolegend	Cat# 301002, RRID:AB_314120
147 - CD45RA (clone HI100)	Biolegend	Cat# 304102, RRID:AB_314406
147 - CD150 (clone A12)	Biolegend	Cat# 306302, RRID:AB_314590
147 - CD4 (clone RPA-T4)	Biolegend	Cat# 300502, RRID:AB_314070
148 - CD45RO (clone UCHL1)	Biolegend	Cat# 304202, RRID:AB_314418
149 - CCR6 (clone G034E3)	Biolegend	Cat# 353402, RRID:AB_10918625
150 - CD103 (clone B-Ly7)	Thermofisher	Cat# 14-1038-80, RRID:AB_467411
151 - KLRG1 (clone 13F12F2)	Thermofisher	Cat# 16-9488-85, RRID:AB_2637116
152 - 2B4 (clone C1.7)	Biolegend	Cat# 329502, RRID:AB_1279194
152 - ICOS (clone C398.4A)	Biolegend	Cat# 313502, RRID:AB_416326
153 - CD25 (clone M-A251)	Biolegend	Cat# 356102, RRID:AB_2561752
154 - CCR4 (clone MAB1567_100)	R&D	Cat# MAB1567, RRID:AB_2074395
154 - CD186 (clone K041E5)	Biolegend	Cat# 356002, RRID:AB_2561738
155 - Tbet (clone eBio4B10)	Thermofisher	Cat# 14-5825-82, RRID:AB_763634
156 - GATA3 (clone TWAJ)	Thermofisher	Cat# 14-9966-82, RRID:AB_1210519
157 - TIM3 (clone F38-2E2)	Biolegend	Cat# 345002, RRID:AB_2116574
158 - CD38 (clone HIT2)	Biolegend	Cat# 303502, RRID:AB_314354
159 - CD161 (clone HP-3G10)	Biolegend	Cat# 339902, RRID:AB_1501090
160 - PD-1 (clone eBioJ105)	Thermofisher	Cat# 14-2799-80, RRID:AB_763476
161 - PLZF (clone MAB2944)	R&D	Cat# MAB2944, RRID:AB_10718564
162 - CX3CR1 (clone K0124E1)	Biolegend	Cat# 355702, RRID:AB_2561726
162 - CXCR3 (clone 49801)	R&D	Cat# MAB160, RRID:AB_2086754
162 - TNFa (clone MAB11)	Biolegend	Cat# 502902, RRID:AB_315254
163 - il7r (clone AO19D5)	Biolegend	Cat# 351302, RRID:AB_10718513
164 - VD1 FITC (clone REA173)	Miltenyi	Cat# 130-100-534, RRID:AB_2653951
165 - Va7.2 (clone 3C10)	Biolegend	Cat# 351702, RRID:AB_10900258
166 - CCR9 (clone L053E8)	Biolegend	Cat# 358902, RRID:AB_2562298
166 - NKP46 (clone 195314)	R&D	Cat# MAB1850, RRID:AB_2149153
166 - CCR5 (clone HEK/1/85a)	Abcam	N/A
166 - CD107a (clone H4A3)	Biolegend	Cat# 328602, RRID:AB_1134259
167 - gd PE (clone B1)	Biolegend	Cat# 331210, RRID:AB_1089218
168 - CCR7 (clone 150503)	R&D	Cat# MAB197, RRID:AB_2072803
168 - il17a (clone BL168)	Biolegend	Cat# 512302, RRID:AB_961399

(*Continued on next page*)

CellPress
OPEN ACCESS

Continued

REAGENT or RESOURCE	SOURCE	IDENTIFIER
169 - CD85J (clone GHI/75)	Biolegend	Cat# 333702, RRID:AB_1089089
169 - HELIOS (clone 22F6)	Biolegend	Cat# 137202, RRID:AB_10900638
171 - VD2 (clone B6)	Biolegend	Cat# 331402, RRID:AB_1089226
172 - VG9 (clone B3)	Biolegend	Cat# 331301, RRID:AB_1089235
173 - vd1 apc (clone REA173)	Miltenyi	Cat# 130-100-519, RRID:AB_265395
174 - CD160 (clone 688327)	R&D	Cat# MAB6700, RRID:AB_10891689
174 - TIGIT (clone MAB7898)	R&D	Cat#MAB7898
174 - CTLA4 (clone BNI3)	BD	Cat# 555851, RRID:AB_396174
174 - granzyme B (clone CLB-GB11)	Thermofisher	Cat# MA1-10338, RRID:AB_11154492
175 FOXP3 BIOTIN (clone PCH101)	Thermofisher	Cat# 13-4776-82, RRID:AB_763539
175 perforin (clone B-D48)	Abcam	Cat# ab47225, RRID:AB_2169084
176 - CD19 (clone HIB19)	Biolegend	Cat# 302202, RRID:AB_314232
176 - CD39 (clone A1)	Biolegend	Cat# 328202, RRID:AB_940438
176 - CD26 (clone BA5b)	Biolegend	Cat# 302702, RRID:AB_314286
209 - CD16 (clone 3G8)	Fluidigm	Cat# 3209002B, RRID:AB_2756431
CD161 (clone HP-3G10)	Biolegend	Cat# 339916, RRID:AB_2563607
CD3 (clone OKT3)	Biolegend	Cat# 317324, RRID:AB_2563352
CD45 (clone HI30)	Biolegend	Cat# 304042, RRID:AB_2562106
CD39 (clone A1)	Biolegend	Cat# 328212, RRID:AB_2099950
CD4 (clone RPA-T4)	Biolegend	Cat# 300546, RRID:AB_2563314
CD8 (RPA-T8)	Biolegend	Cat# 301033, RRID:AB_1595443
IFNg (clone 4S.B3)	Thermofisher	Cat# 11-7319-82, RRID:AB_465415
TNFa (clone MAb11)	BD	Cat# 560679, RRID:AB_1727579
IL17 (clone N49-653)	BD	Cat# 563745, RRID:AB_2738401
Va7.2 (clone 3C10)	Biolegend	Cat# 351716, RRID:AB_2563864
7-AAD	Thermofisher	Cat#A1310
Annexin V	Biolegend	Cat#640906
PI	Thermofisher	Cat#P1304MP
FLICA	Thermofisher	Cat# V35118
APC streptavidin	Biolegend	Cat#405207
PE streptavidin	Biolegend	Cat#405204
Bacterial and Virus Strains		
Bacteriodes thetaiotamicron	DSM	2079 (VPI 5482)
Bacteroides vulgatus	ATCC	8482
Fusobacterium nucleatum	ATCC	25586
Escherichia coli	Invitrogen	18265017
Biological Samples		
CRC tumor, healthy tissue and blood samples	Singapore	N/A
NSCLC tumor, healthy tissue and blodd samples	Singapore	N/A
RCC tumor, healthy tissue and blood samples	North-West BiosTrust	N/A
Chemicals, Peptides, and Recombinant Proteins		
MR1 tetramer	NIH tetramer core	N/A
CD1d tetramer	NIH tetramer core	N/A
Critical Commercial Assays		
Rhapsody AbSeq reagent pack	BD Biosciences	Cat#633771
Rhapsody Human T cell expression panel	BD Biosciences	Cat#633751
Human Single cell multiplexing kit	BD Biosciences	Cat#633781

(*Continued on next page*)

Continued		
REAGENT or RESOURCE	SOURCE	IDENTIFIER
Deposited Data		
Sc-RNaseq/AbSeq data	https://www.ncbi.nlm.nih.gov/geo/	GEO: GSE151842
Experimental Models: Cell Lines		
THP-1	ATCC	TIB-202
Software and Algorithms		
R Studio and R environment	The R project for Statistical Computing	https://rstudio.com/ and https://cran.r-project.org/
Seven Bridges (pre-processing of Rhapsody FASTQ files)	BD Biosciences	https://www.sevenbridges.com

RESOURCE AVAILABILITY

Lead Contact

Further information and requests for resources and reagents should be directed to and will be fulfilled by the Lead Contact, Evan W. Newell (enewell@fredhutch.org).

Materials Availability

This study did not generate new unique reagents.

Data and Code Availability

The accession number for the scRNAseq data discussed in this study is GSE15184 at NCBI's Gene Expression Omnibus.

EXPERIMENTAL MODEL AND SUBJECT DETAILS

Human samples

Blood, tumors and adjacent tissues samples were collected from patients with Colorectal Cancer (CRC), Non-Small Cell Lung Cancer (NSCLC) or Renal cell carcinoma (RCC). The use of human tissues was approved by the appropriate institutional research boards, A*STAR and the Singapore Immunology Network, Singapore. The RCC samples were provided by Northwest Biotrust, under a NWBiospecimens protocol, Seattle. NW BioTrust, a core service for patient consenting, and NWBioSpecimen, a core service for procurement and annotation of research biospecimens, are supported by National Cancer Institute grant P30 CA015704 (G. Gilliland, principal investigator [PI]), Institute of Translational Health Sciences grant UL1 TR000423 (M. Disis, PI), the University of Washington School of Medicine and Department of Pathology, and Fred Hutchinson Cancer Research Center. The analysis was performed according to the IRB file/approval number NHS #6007-1061. No age or gender information is available. Informed written consent was obtained from each subject or each subject's guardian.

Bacterial strains

DH5α *E. coli* was aerobically cultured overnight in a shaking incubator at 37°C in Luria-Bertani broth. *Bacteroides thetaiotaomicron* and *Bacteroides vulgatus* were anaerobically cultured for 18-24 hours in modified gut microbiota media as previously described.[53] *Fusobacterium nucleatum* was anaerobically cultured for 18-24 hours in fastidious anaerobic broth as previously described.[54] Bacterial growth of all strains was estimated by measuring optical densities at 600 nm (OD_{600}).

METHOD DETAILS

Cell isolation

Samples were prepared as previously described.[22] Briefly, tissues were mechanically dissociated into small pieces and incubated at 37°C for 10-20min in DMEM + collagenase IV (1mg/ml) + DNase (10ug/ml). Digestion was stopped by addition of RPMI+5% FBS. Dissociated tissues were filtered and washed twice before cryopreservation in freezing medium (90% FBS and 10% DMSO). PBMCs from patients and healthy donors were isolated from peripheral blood samples by Ficoll separation and washed twice with PBS before cryopreservation.

CyTOF staining

Purified antibodies lacking carrier proteins were purchased according to the provided in the Key Resource Table. Antibody conjugation was performed according to the protocol provided by Fluidigm. Streptavidin was labeled as previously described.[55]

MR1- and CD1d-tetramers are provided by the NIH Tetramer Core Facility and were tetramerized as stated with our metal-labeled streptavidins. Frozen samples were thawed and washed twice in thawing medium (RPMI, 10% FBS, 10 μg/ml DNase). Cells were first enriched for CD45 using purified anti-CD45 antibody followed by anti-mouse IgG microbeads, and positively selected using MACS LS columns. Cells were then stained for cisplatin (5 μM) in PBS for 5 min on ice, washed and incubated for 1h at room temperature with MR1-tetramer. Cells were then incubated with primary and secondary surface antibodies cocktails for 20min each on ice, fixed and permeabilized using Foxp3 transcription factor staining buffer set (eBioscience) and stained for primary and secondary intranuclear antibodies for 30min each on ice. Cells were then washed three times and fixed overnight in PFA 2%.

The next day, the cells were washed twice before barcoding with in-house barcodes in PBS as previously described for 30 min at 4°C,[56] washed and stained for DNA for 15 min at room temperature (Cell-ID intercalator-Ir, Fluidigm). Cells were lastly washed three times with dH2O, counted and run on CyTOF at a ratio of 0.5M cells/ml in dH2O.

in vitro MAIT cell stimulation

MAIT cells were stimulated for 16h with either IL-12+IL-18 (R&D) at 50ng/ml or different strains of bacteria as described above. For *E. coli*, bacteria were fixed using 2% PFA and washed extensively in PBS. For *B. thetaiotaomicron, B. vulgatus*, and *F. nucleatum,* either culture supernatant, live or fixed cell pellets were used. Bacterial cells were pelleted by centrifugation (15,000xg for 5 minutes), and any remaining bacterial cells were removed from the supernatant through syringe-filtration (0.2 μM). Next, 1M of growing ThP-1 cells (ATCC® TIB-202) were incubated with 25M of either E.coli or other bacteria in ThP-1 culture medium (RPMI-1640 + 0.05mM of 2-mercaptoethanol and 10% of FBS). 4-5h later, bacteria were washed away and 2M of PBMCs were added to the culture and stimulated for 16h. BFA (eBioscience) was added for the last 5 hours to stop the stimulation. For MR1-blocking assay, purified anti-MR1 antibody (clone 26.5, Biolegend) was added to the culture before the PBMCs and incubated for 20 min at 4°C. The activation of MAIT cells was then measured through IFNγ, TNFα or IL-17a expression by Flow cytometry.

FACS analysis

Frozen samples were thawed as mentioned above and surface stained for FACS using antibodies listed in the Key Resource Table. For Foxp3 staining, cells were fixed and permeabilized using Foxp3 transcription factor staining buffer set, incubated with biotin anti-FoxP3 and then APC streptavidin for 30 min each at 4°C. For apoptosis assay, cells were stained using Vybrant FAM Poly Caspases Assay kit (ThermoFisher) and according to manufacturer's instructions. Samples were run on BD FACSCelesta or LSRFortessa and analyzed using FlowJo.

QUANTIFICATION AND STATISTICAL ANALYSIS

CyTOF data analysis and UMAP

After mass cytometry (CyTOF) acquisition, any zero values were randomized using a uniform distribution of values between 0 and −1 using R. The signal of each parameter was normalized based on EQ beads (Fluidigm) as described previously.[57] Cells were manually debarcoded using FlowJo. Samples were then used for UMAP analysis similar to that previously described using customized R scripts based on the 'flowCore' and 'uwot' R packages.[19] In R, all data were transformed using the logicleTransform function (flowCore package) using parameters: $w = 0.25$, $t = 16409$, $m = 4.5$, $a = 0$ to roughly match scaling historically used in FlowJo. For heatmaps, median intensity corresponds to a logical data scale using formula previously described.[58] The colors in the heatmap represent the measured means intensity value of a given marker in a given sample. A seven-color scale is used with black–blue indicating low expression values, green–yellow indicating intermediately expressed markers, and orange-red representing highly expressed markers.

mRNA sequencing and Gene enrichment analysis

Gene expression profiles of tumor-infiltrating MAIT cells were obtained from FACS-sorted MAIT cells using tet-MR1 and CD161. Paired-end RNA-Seq reads from Illumina HiSeq 4000 were counted based on the human GENCODE V27 gene annotations[59] using Salmon[60] to the gene level. The logarithmically transformed counts were filtered for IQR (Inter Quartile Range) > 0.5. The filtered genes counts were used in edgeR[61] to identify the differentially expressed genes (DEG) on genes. Multiple testing correction was conducted using the method of Benjamini and Hochberg with adjusted P values < 0.05 deemed to be significant. All analyses were done in R v.3.3.3. We ran PCA using the prcomp function from the stats (v.3.4.2) R package, using the whole transcriptomic data. We used the HTSanalyzeR package (v.2.26.0) to run GSEA on gene collections from the Gene Ontology Biological Processes database, filtered for gene sets with at least 20 genes present in our dataset. For GSEA we used 1000 permutations to estimate *P*values and applied corrections for multiple tests using the Benjamini–Hochberg procedure.

BD AbSeq single-cell mRNA sequencing

Tumor-infiltrating MAIT cells from 3 CRC patients and control PBMC from 1 healthy donor were obtained by FACS sorting using tet-MR1. Cells were then incubated with BD AbSeq Ab-oligos following manufacturers' instructions (BD Biosciences). Single cells were isolated using Single Cell Capture and cDNA synthesis with the BD Rhapsody Express Single-cell Analysis System. Parallel RNA and BD AbSeq sequencing libraries were generated using BD Rhapsody targeted mRNA and AbSeq amplification and BD Single-cell

Multiplexing kits and protocol. Quality of final libraries was assessed using Agilent 2200 TapeStation with High Sensitivity D5000 ScreenTape, quantified using a Qubit Fluorometer (ThermoFisher), and carried through to sequencing with Novaseq S1 on Illumina sequencer. FASTQ files containing sequenced data were analyzed using the Seven Bridges platform provided by BD (See "BD Single Cell Genomics Bioinformatics Handbook – 54169 Rev. 6.0" for specific details).[20] The output files containing proteins and genes expression for each cell were transformed using log2 with a pseudo count of 1 and analyzed for differentially expressed genes or proteins using the *FindMarkers* function of the Seurat (v2.3.4) R package[62] using a Wilcoxon test (default) as differential expression method and an expression frequency threshold of 0.

DNA extraction, WGS and metagenomic analysis

DNA Sequencing was performed as previously reported.[63] In brief, ten 5-um tissue sections were used to obtain tissue for whole genome sequencing (WGS). DNA-Seq libraries were prepared using the KAPA Hyper Prep Kit (Kapa Biosystems, Wilmington, MA) and sequenced on the Illumina HiSeq platform (Illumina, San Diego, CA). Raw sequencing reads were aligned to the human genome (hg19 assembly) using the BWA aligner.[64] Reads that were not mapped to the human genome based on this strict criterion were assigned to bacterial species using the Kraken software[33] with the MiniKraken database and default parameters.

Expression of riboflavin genes in CRC metagenome

Violin plot of the estimated coefficients in CRC versus healthy patient metagenome for different microbiome-encoded genes involved in riboflavin metabolism (grouped by their enzyme commission (EC) number). Analysis performed with data published in Minot et al., Microbiome, 2019.[43] A positive number indicates that the gene is enriched in the CRC metagenome, whereas a negative number indicates that the gene is enriched in the healthy metagenome. The first plot is for all genes except the riboflavin metabolism genes. Fisher's exact test was used for each riboflavin gene to assess for association with cancer compared to all other genes.

Cell Reports Medicine, Volume *1*

Supplemental Information

Human Tumor-Infiltrating MAIT Cells Display Hallmarks of Bacterial Antigen Recognition in Colorectal Cancer

Shamin Li, Yannick Simoni, Etienne Becht, Chiew Yee Loh, Naisi Li, Daniel Lachance, Si-Lin Koo, Teck Por Lim, Emile Kwong Wei Tan, Ronnie Mathew, Andrew Nguyen, Justin Golovato, Julia D. Berkson, Martin Prlic, Bernett Lee, Samuel S. Minot, Niranjan Nagarajan, Neelendu Dey, Daniel S.W. Tan, Iain B. Tan, and Evan W. Newell

Supplementary Figure S1

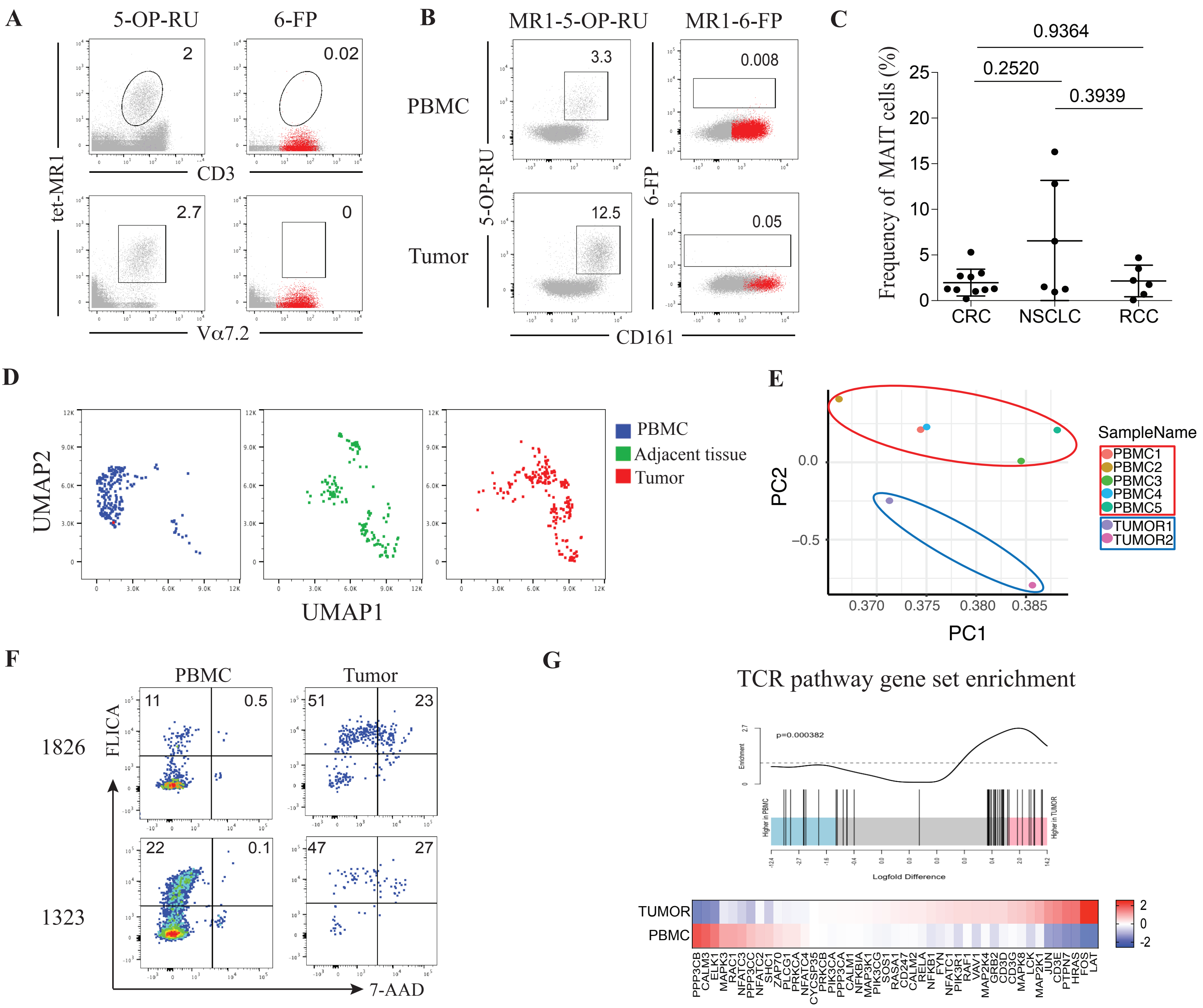

Supplementary figure S1: Tumor-infiltrating MAIT cells express more apoptotic signals and a higher TCR signaling, related to Figure 1. **A**. Representative staining of MAIT cells from HD PBMC by mass cytometry, as gated on MR1-tet loaded with 5-OP-RU or 6-FP (negative control), along with CD3 on total live CD45$^+$CD14$^-$CD16$^-$ cells (upper panel), with Vα7.2 on total CD45$^+$CD14$^-$CD16$^-$CD3$^+$T cells (lower panel). The MR1-6-FP$^+$ cells are shown in purple on the left panel, the MR1-5-OP-RU$^+$CD161$^+$ cells are shown in red on the right panel. **B.** Representative tet-MR1 staining (5-OP-RU and control 6-FP) gated on CD45$^+$CD3$^+$ T cells from healthy donor (HD) PBMC or CRC tumor by flow cytometry. The MR1-6-FP$^+$ cells are shown in purple on the left panel, the MR1-5-OP-RU$^+$CD161$^+$ cells are shown in red on the right panel. **C.** Frequency of total MAIT cells in PBMC of CRC (n=10), NSCLC (n=6), RCC (n=6) patients. Data are mean with S.D. from at least 7 experiments. Mann-Whitney U test. **D**. Individual UMAP plot analyzing MAIT cell phenotype from each compartment. **E**. PCA analysis of the transcriptomic profile of MAIT cells from tumors (blue circle) as compared to PBMCs (red circle). Tumors and PBMCs are paired for the samples 1 and 2. **F**. Representative staining showing MAIT cell apoptosis in two tumors and paired PBMCs, as assessed by FLICA (caspase activation) and 7-AAD staining. The numbers are patients ID. **G.** Gene Set Enrichment Analysis of MAIT cells in tumors as compared to the paired PBMCs, based on the genes from the TCR pathway (upper panel). Heatmap of genes that are differentially expressed in MAIT cells between tumors and PBMCs (lower panel). The colors indicate the expression intensities, from low (blue) to high (red).

Supplementary figure S2

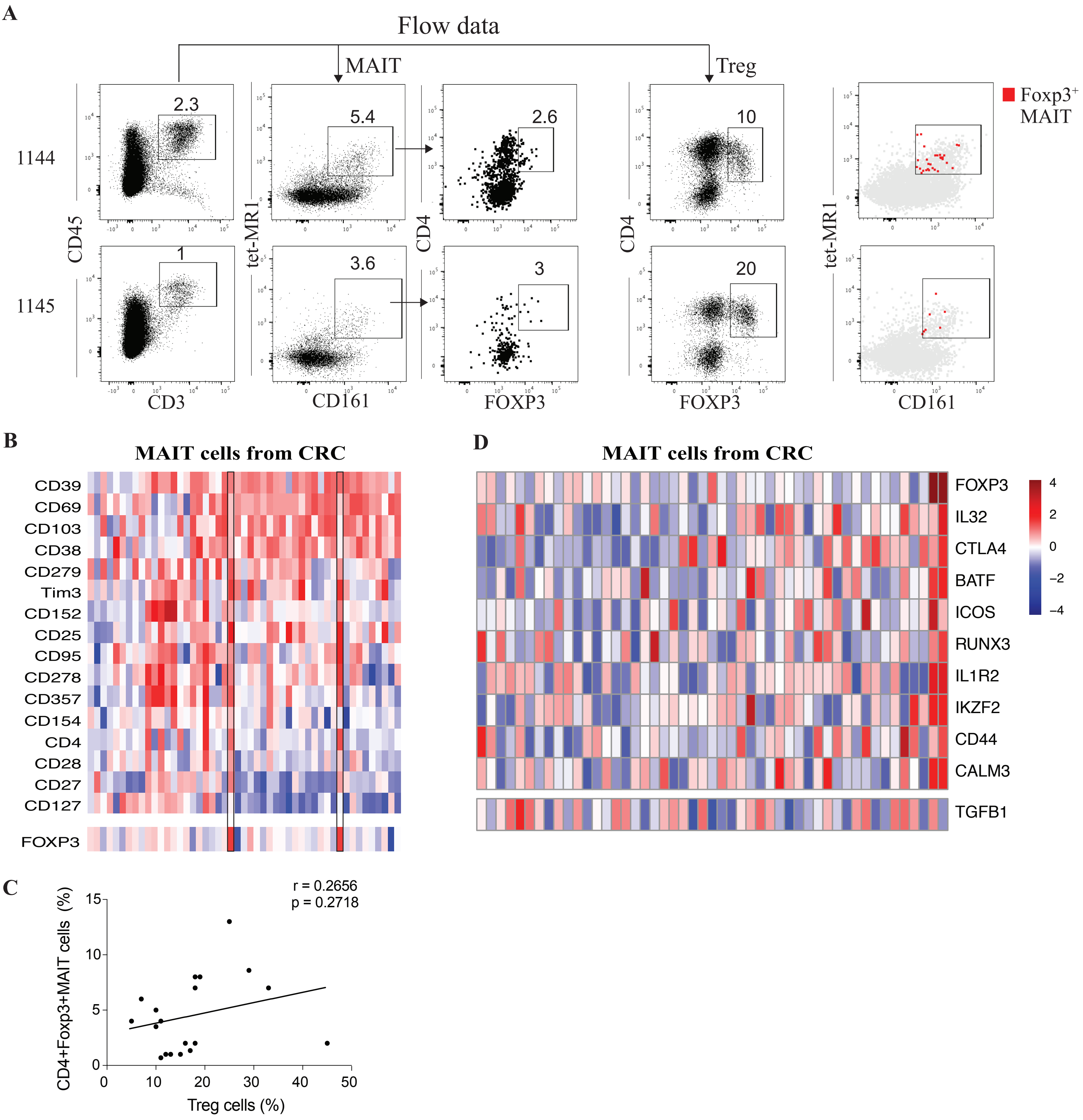

Supplementary figure S2: Tumor-infiltrating CD4$^+$Foxp3$^+$MAIT cells in CRC, related to Figure 2.

A. Representative flow cytometry staining of CD4$^+$Foxp3$^+$MAIT cells in two colorectal tumor samples, as compared with conventional Treg cells from the same tumor (left panel). Backgating of CD4$^+$Foxp3$^+$MAIT cells (red) on total MAIT cells on the same samples (right panel). **B.** Heatmap showing expression intensities of selected surface proteins vs. FOXP3 in MAIT cells from CRC tumor. Single-cell targeted mRNAsequencing combined with BD ABseq Rhapsody system on sorted MAIT cells (see methods). **C.** Correlation of Foxp3 expression on MAIT cells with Treg cells, n=20. **D.** Heatmap showing expression intensities of selected activation genes and TGFB1 vs. FOXP3 in tumor-infiltrating MAIT cells.

Supplementary figure S3

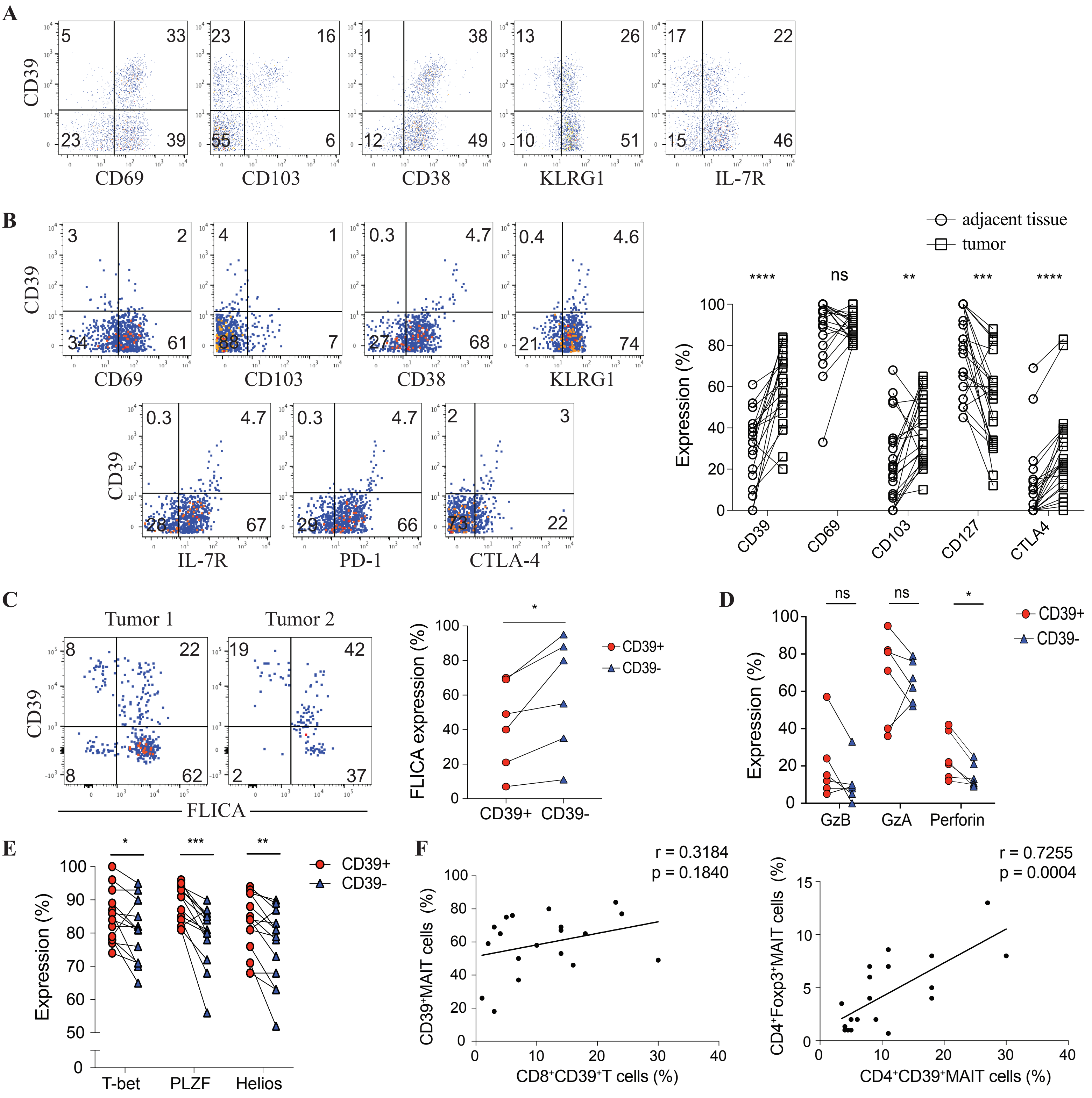

Supplementary figure S3: Tumor-infiltrating CD39$^+$MAIT cells in CRC, related to Figure 3.

A. Coexpression of selected markers with CD39 on tumor-infiltrating MAIT cells from one representative CRC patient. **B.** Coexpression of selected markers with CD39 on MAIT cells of adjacent colon tissue from one representative CRC patient (left panel). Comparison of selected markers expression between MAIT cells from adjacent tissue and tumor (right panel), n=19-22, Two-tailed paired t-test. **C.** Expression of FLICA (caspase activation) and CD39 on tumor-infiltrating MAIT cells. n=6. Data are from 2 experiments. Two-tailed paired t-test. **D.** Expression of Granyme B, Granzyme A and perforin on CD39$^+$ vs. CD39$^-$ tumor-infiltrating MAIT cells upon PMA/Iono stimulation. n=6. Data are from 2 experiments. Two-tailed paired t-test. **E.** Expression of T-bet, PLZF and Helios on CD39$^+$ vs. CD39$^-$ tumor-infiltrating MAIT cells, n=14. Data are from at least 3 experiments. Two-tailed paired t-test. **F.** Correlation of CD39 expression on MAIT cells with CD8$^+$CD39$^+$T cell frequencies, CD4$^+$Foxp3$^+$MAIT cells with CD4$^+$CD39$^+$MAIT cell frequencies, n=20.

Supplementary Figure S4

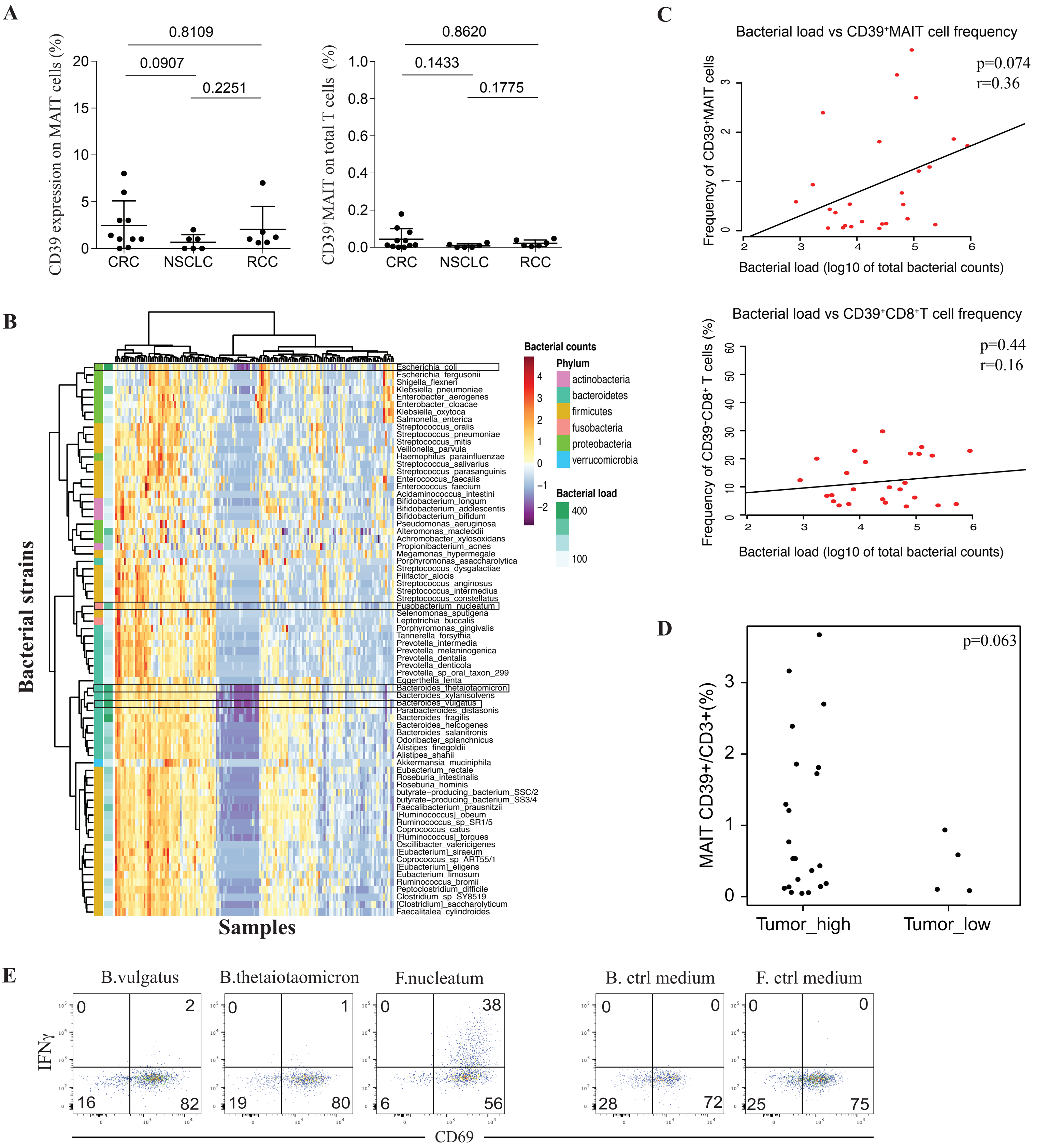

Supplementary figure S4: Extended figures of the microbiome-related analysis, related to Figure 4.

A. CD39 expression on MAIT cells (left) and on total T cells (right) in PBMC of CRC(n=10), NSCLC(n=6), RCC(n=6). Data are mean with S.D. from at least 7 experiments. Mann-Whitney U test. **B.** Bi-clustering of tumor samples (n=155) vs. selected bacterial species (n=74) after prefiltering. The number of species was cut down from 1479 to 74 based on the bacterial load across all tumor samples (>100). Each row represents one bacterial species, each column one tumor sample. The bacteria species were also classified according to the phylum they belong to (see legends). **C.** Correlation of relative bacterial load summed across all species for each tumor with CD39^{+}MAIT cell frequency on total CD3^{+}T cells (upper panel) or CD39^{+}CD8^{+}T cell frequency on total CD3^{+}T cells (lower panel), n=26. **D.** CD39^{+}MAIT cell frequency in tumors that are highly (n=22) or hardly bacteria-infiltrated (n=4). p=0.063, non-parametric t-test. **E.** *In vitro* screening of *Bacteroides* and *Fusobacterium* strains in their capability to activate MAIT cells. Culture media are used as controls.

Supplementary Figure S5

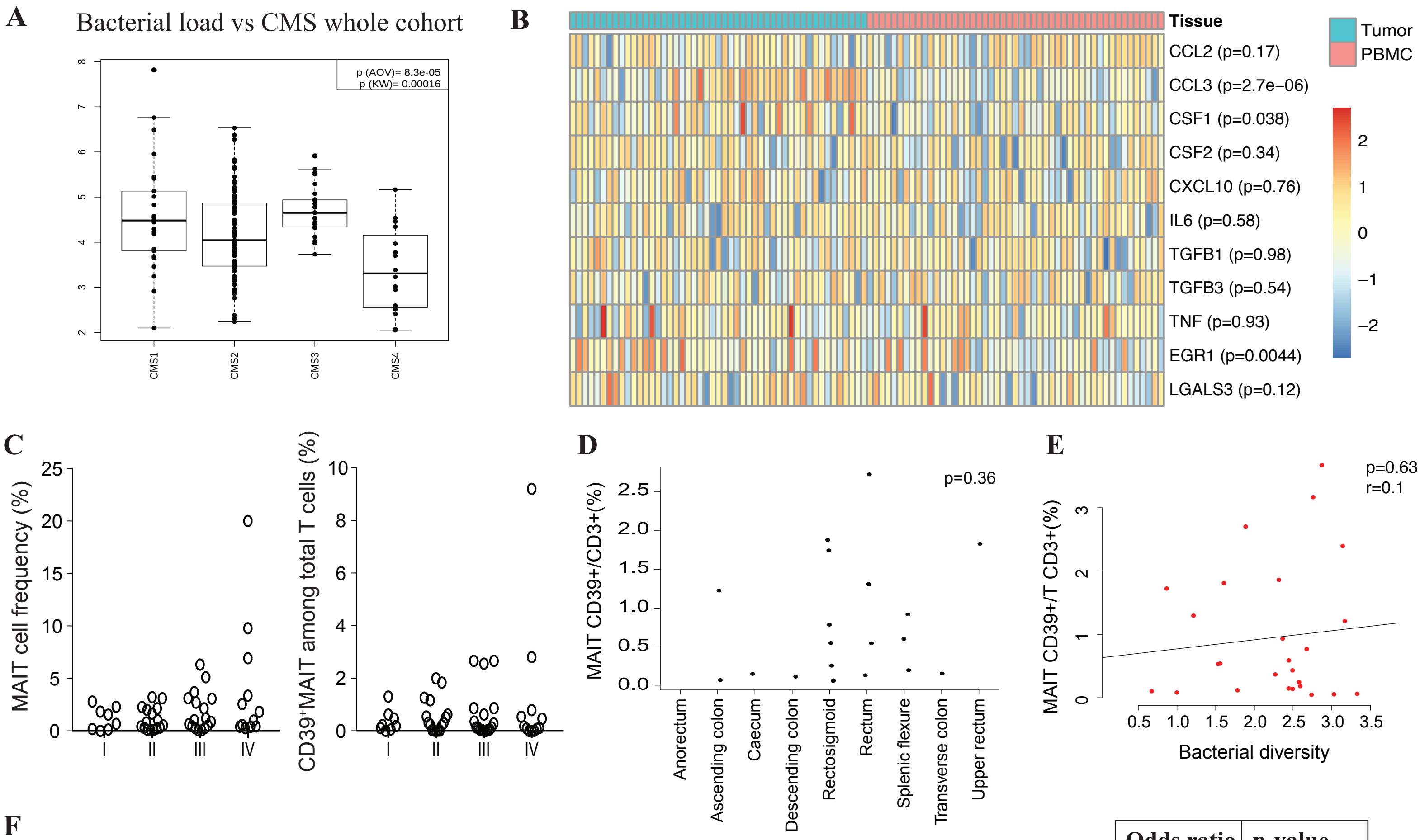

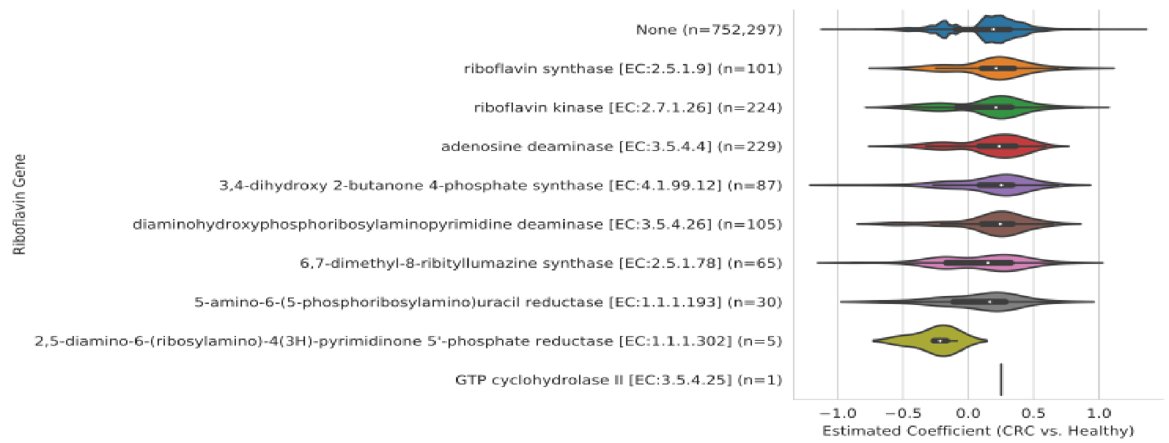

Odds ratio	p-value
-	-
1.47	0.12
1.08	0.66
1.35	0.07
1.21	0.48
**2**	**0.0063**
0.62	0.07
0.77	0.54
0.00	0.0017
-	-

Supplementary figure S5: Clinical associations in CRC, related to Figure 4.

A. Integration of the bacterial load obtained for each sample of the cohort to the consensus molecular classification of CRC previously described (36, 37). **B.** Heatmap of tissue-repair associated genes from scRNAseq profiling of MAIT cells in CRC tumor (blue) vs. healthy donor PBMC (red). Wilcoxon Mann-Whitney test. **C.** Frequency of total MAIT and $CD39^+$MAIT on total T cells in CRC tumor samples according to the disease stage (I, II, III or IV). n=8 for stage I, 16 for stage II, 17 for stage III and 12 for stage IV. **D**. Association between $CD39^+$MAIT cell frequency and tumor localization, n=21. **E.** Correlation between bacterial diversity and $CD39^+$MAIT cell frequency among total $CD3^+$ T cells, n=26. **F.** Violin plot showing distributions of the estimated coefficients in CRC vs. healthy metagenome for different microbiome-encoded genes involved in riboflavin metabolism. Analysis performed from data of *Minot et al, Microbiome, 2019* (40) (see methods).

Data S1

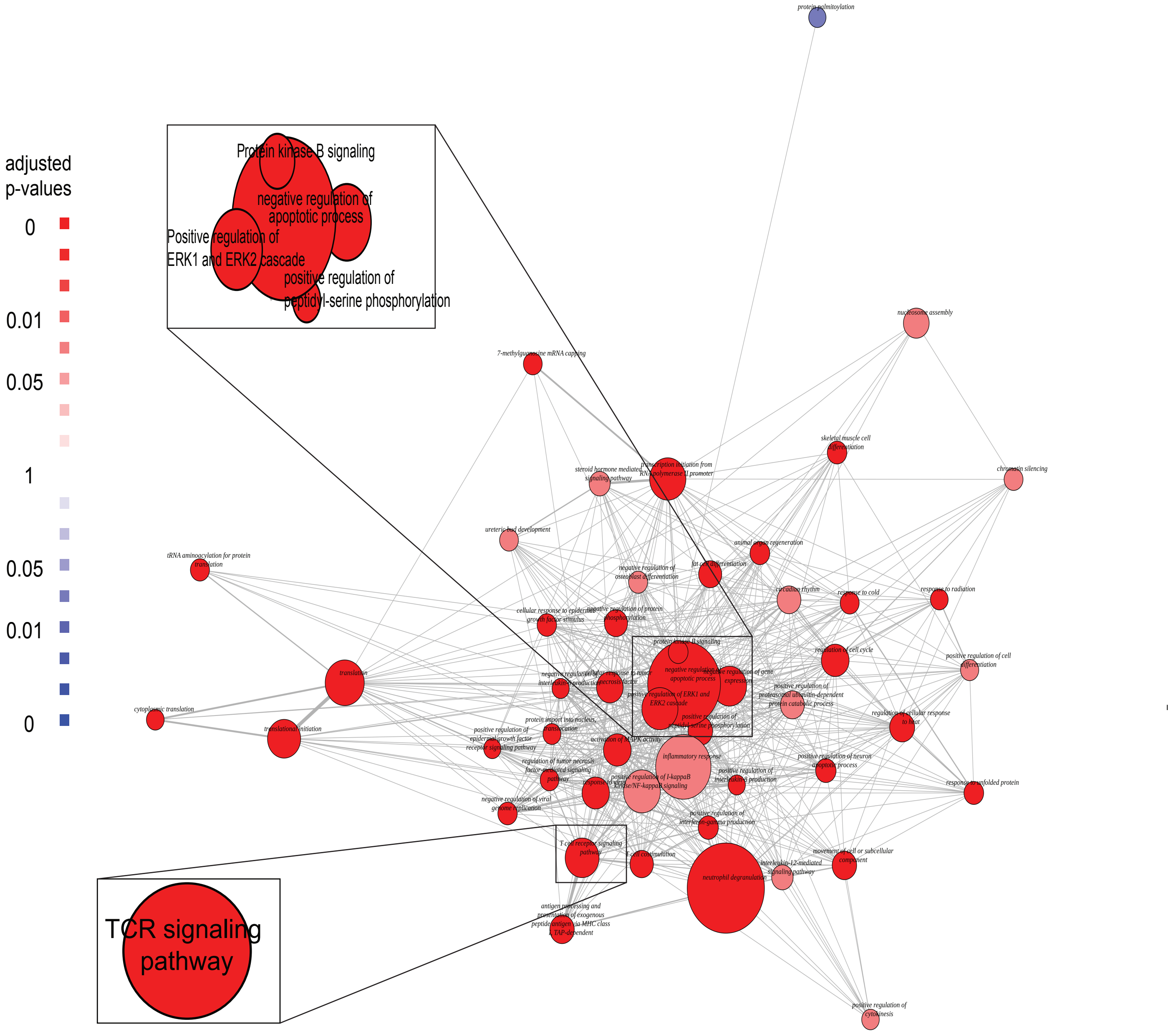

Data S1: Tumor-infiltrating MAIT cells express more apoptotic signals and a higher TCR signaling, related to Figure 1.

Gene enrichment map of MAIT cells in the tumors as compared to the paired PBMCs (n=2).
